# NIR‐Emissive Chromium(0), Molybdenum(0), and Tungsten(0) Complexes in the Solid State at Room Temperature

**DOI:** 10.1002/chem.202102208

**Published:** 2021-08-04

**Authors:** Pit Boden, Patrick Di Martino‐Fumo, Tobias Bens, Sophie Steiger, Uta Albold, Gereon Niedner‐Schatteburg, Markus Gerhards, Biprajit Sarkar

**Affiliations:** ^1^ Department of Chemistry and Research Center Optimas TU Kaiserslautern Erwin-Schrödinger-Straße 52 67663 Kaiserslautern Germany; ^2^ Chair of Inorganic Coordination Chemistry Institute of Inorganic Chemistry University of Stuttgart Pfaffenwaldring 55 70569 Stuttgart Germany; ^3^ Institute of Chemistry and Biochemistry Freie Universität Berlin Fabeckstraße 34–36 14195 Berlin Germany

**Keywords:** carbonyl ligands, mesoionic carbenes, NIR II emitters, step-scan FTIR spectroscopy, X-ray diffraction

## Abstract

The development of NIR emitters based on earth‐abundant elements is an important goal in contemporary science. We present here Cr(0), Mo(0), and W(0) carbonyl complexes with a pyridyl‐mesoionic carbene (MIC) based ligand. A detailed photophysical investigation shows that all the complexes exhibit dual emissions in the VIS and in the NIR region. The emissive excited states are assigned to two distinct triplet states by time‐resolved emission and step‐scan FTIR spectroscopy at variable temperature, supported by density functional theory. In particular, the NIR emissive triplet state exhibits unprecedented lifetimes of up to 600±10 ns and quantum yields reaching 1.7 ⋅ 10^−4^ at room temperature. These are the first examples of Cr(0), Mo(0) and W(0) complexes that emit in the NIR II region.

## Graphical abstract

**This work presents** emissive Cr(0), Mo(0), and W(0) complexes that contain a bidentate pyridyl‐mesoionic carbene ligand and CO coligands. These systems exhibit dual vis and NIR II emissions at room temperature. The emissive excited triplet states were characterized by temperature‐dependent luminescence and step‐san FTIR spectroscopy in combination with density functional theory. The NIR quantum yield increases from Cr(0) over Mo(0) to W(0), reaching a very high value of 1.4 ⋅ 10^−3^ at room temperature.

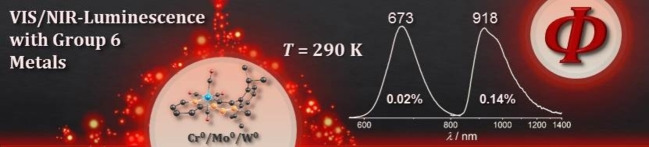

The development of noble metal free highly luminescent molecular systems that do not contain precious rare earth elements like for example iridium(III), platinum(II) and lanthanides is an important and rapidly growing research field.^[1]^ Most of the systems based on earth‐abundant metals reported so far feature a visible emission.^[2]^ In this context, copper(I) systems have already turned out as suitable luminophores in noble metal free organic light emitting diodes (OLEDs).^[3]^ However, there is only a very limited number of near‐infrared (NIR) emissive systems based on earth‐abundant metals.[^4^, ^5^] The design and synthesis of such molecular NIR emitters is a rapidly growing research field due to the broad area of applications covering the fields of telecommunication^[6]^, OLED^[7]^ and LEC^[8]^ devices as well as bioanalysis and bioimaging.^[9]^

More than four decades ago Mann et al. reported luminescent octahedral chromium(0), molybdenum(0) and tungsten(0) complexes with arylisocyanide ligands (M(CNR)_6_, R=phenyl or 2,6‐diisopropylphenyl) showing a visible emission in solution at room temperature.^[10]^ In the last years, the group of Gray published W(0) systems with microsecond lifetimes by tuning the arylisocyanide ligands and in particular pushed the luminescence decay time to 3.83 μs in toluene at room temperature by extending the π ‐system.[^11^, ^12^] Furthermore, the luminescence of the W(0) complexes with expanded ligands reaches into the NIR I region (λ_em_=780–1000 nm).[^11^, ^12^] In the last years the Wenger group developed related complexes with sterically demanding bidentate isocyanide ligands.[^13^, ^14^, ^15^] A long lifetime of 1.1 μs was achieved with a Mo(0) complex in toluene at room temperature.^[14]^ Very recently, the emission was red‐shifted by tuning the chelating isocyanide ligands with luminescence spectra tailing into the NIR I region.^[15]^ Next to the design of Cr(0), Mo(0) and W(0) isocyanide complexes, Lees et al. reported in 1982 on Mo(0) and W(0) carbonyl complexes with substituted pyridine ligands (M(CO)_5_L with M=Mo, W; L=substituted pyridine), which are emissive in solution at room temperature.^[16]^ At the same time heteroleptic M(CO)_4_α‐diimine complexes were synthesized, which show a room temperature emission reaching into the NIR I region.[^17^, ^18^, ^19^] Furthermore, phosphorescent W(0) carbonyl pyridyl‐imidazole complexes with luminescence lifetimes of up to 350 ns^[20]^ as well as luminescent polynuclear cluster complexes like for example (Bu_4_N)_2_[Mo_6_I_8_(NO_3_)_6_]^[21]^ have been reported.

The reports on molecular Cr, Mo and W systems with an emission in the NIR II region (λ_em_=1000–1700 nm) are still very rare. In 1988, an NIR emission in solution was reported for the anions MoCl_6_^3−^ and Mo(NCS)_6_^3−^ for the first time.^[22]^ Ten years later Mohammed et al. reported the complexes (Me_3_[9]aneN_3_)Mo(III)X_3_ (X=Cl, Br, I) (Me_3_[9]aneN_3_=1,4,7‐trimethyl‐1,4,7‐triazacyclononane) with an NIR emission in solution at room temperature with emission maxima at 1120–1160 nm and quantum yields of 10^−5^–10^−4^.^[5]^ The longest luminescence wavelengths were, however, achieved for the solid samples of MoCl_3_(py)_3_ and (Me_3_[9]aneN_3_)W(III)Cl_3_ with emission maxima around 1400 nm.^[5]^ There are, up to now, no NIR II emissive complexes of Cr(0), Mo(0) and W(0) to the best of our knowledge. In recent years, mesoionic carbene (MIC) complexes of the 1,2,3‐triazol‐5‐ylidene type were established as privileged ligands for generating emissive transition metal complexes, such as MIC complexes of Fe(II/III),^[23]^ Cu(I),[^24^, ^25^] Ru(II),[^26^, ^27^] Pd(II),^[28]^ Re(I),^[29]^ Os(II),^[27]^ Ir(III),^[30]^ Pt(II)/Pt(IV)^[31]^ and Au(I).[^24^, ^32^]

Here, we present the first Cr(0), Mo(0) and W(0) complexes with an NIR II emission in the solid state at room temperature. These complexes exhibit a second emission in the visible region. The slightly distorted octahedral systems contain the bidentate pyridyl‐MIC ligand (PyC‐NMIC‐dipp) (**L1**) and CO coligands (Figure 1). This work deals with the investigation of the photophysical properties of the three complexes, with a focus on the dual emission, and in particular the NIR II emission.

**Figure 1:**
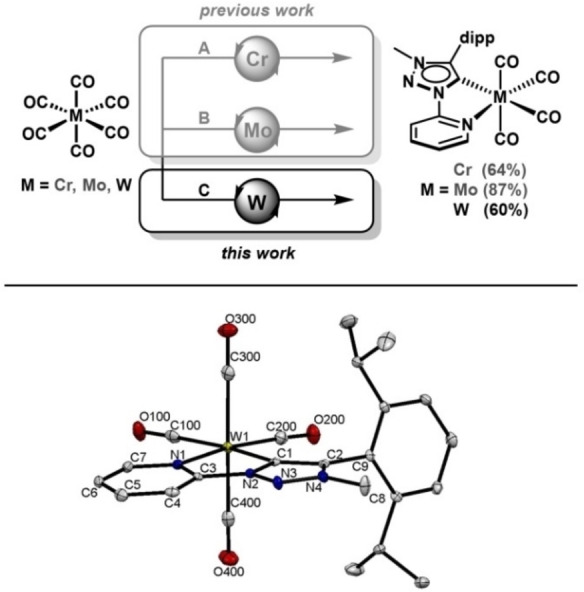
Molecular structure and synthesis of the investigated complexes [M(PyC‐NMIC‐dipp)(CO)_4_] with M=Cr(0), Mo(0)^[33]^ and W(0) (top) as well as ORTEP representation of **W** (bottom). Ellipsoids are drawn with 50 % probability. Hydrogen atoms are omitted for clarity. A: 1) hν , THF, r.t., 2 h; 2) [**HL1**]OTf, NEt_3_, reflux, overnight; B: 1) hν , THF, r.t., 2 h; 2) norbornadiene, reflux, 2 d; 3) [**HL1**]OTf, NEt_3_, r.t., overnight; C: 1) hν , CH_3_CN, r.t., 2 h; 2) [**HL1**]OTf, NEt_3_, reflux, 3 d.

The synthesis of the studied systems [Cr(PyC‐NMIC‐dipp)(CO)_4_] (**Cr**) and [Mo(PyC‐NMIC‐dipp)(CO)_4_] (**Mo**) was presented before.^[33]^ The new complex [W(PyC‐NMIC‐dipp)(CO)_4_] (**W**) was synthesized according to related procedures activating W(CO)_6_ under UV‐irradiation in acetonitrile (CH_3_CN, Figure 1) for two hours. The pyridyltriazolium salt [**HL1**]OTf^[33]^ of the corresponding pyridyl‐MIC ligand was added to the in situ generated solvato complex in presence of NEt_3_ as a base. **W** was isolated in good yields (Figure 1) after extraction and further purification via column chromatography.[^33^, ^34^] The crystal structure (Figure 1) shows W−C and W−N bond lengths of 2.186(3) and 2.259(2) Å. These bonds of the metal center to the **L1** donor atoms of the MIC and pyridyl moiety are in between of those observed for **Cr** and **Mo** in the order **Cr**<**W**<**Mo** as a consequence of the ionic radii of the metal centers. The W−C and C−O bond lengths of the carbonyl coligands (for selected bond lengths, see Supporting Information) follow the same trend as described earlier for **Cr** and **Mo**.^[33]^ Deposition Number 2064469 contains the supplementary crystallographic data for this paper. These data are provided free of charge by the joint Cambridge Crystallographic Data Centre and Fachinformationszentrum Karlsruhe Access Structures service.

The UV/VIS absorption spectra in solution (toluene, dichloromethane and acetonitrile) at room temperature show two low energy bands in the spectral region of 350–600 nm (Figures S4–S6, Supporting Information), which could be assigned to metal to ligand charge transfer transitions according to time‐dependent density functional theory (TDDFT) calculations (see explanations in the Supporting Information, Figures S7–S9, S37–S39, Tables S6–S8). The position of these low energy bands is dependent on the metal center and the solvent (Figures S4–S6, Supporting Information). The lowest energy absorption is observed for **Cr**, the highest for **Mo**, and **W** is lying in between, this trend being independent of the solvent. Furthermore, large blue‐shifts of up to about 70 nm are observed for these low energy transitions by increasing solvent polarity from toluene over dichloromethane to acetonitrile. This is an experimental proof that these transitions show charge transfer character, in accordance with the abovementioned TDDFT calculations. Additionally, these TDDFT calculations confirm the spectral shifts observed upon exchange of the metal center and variation of the solvent polarity. Solvent effects were considered by using the conductor‐like screening model (COSMO). The large impact of solvent polarity was observed for related M(CO)_4_L (M=Cr, Mo, W, L=diimine) complexes before.[^19^, ^20^, ^35^] Furthermore, the UV/VIS absorption spectra in the solid state (KBr pellets) are even further red‐shifted with onsets between 633 and 686 nm following the trend **Mo**<**W**<**Cr** observed already in solution (Figure 2). This red absorption led us to thorough photoluminescence investigations, especially with respect to a potential NIR emission.

**Figure 2:**
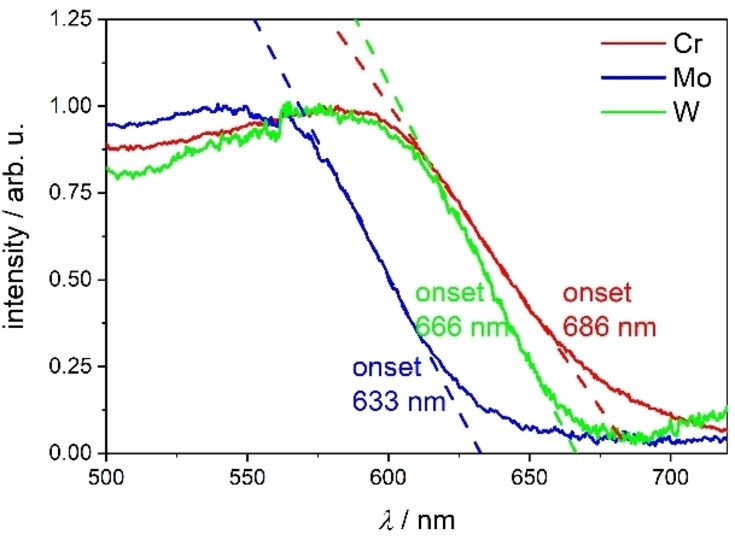
Solid state UV/VIS absorption spectra (KBr pellets) of **Cr**, **Mo** and **W**. The absorption onsets were approximated tangentially.

The visible (VIS) emission spectra of KBr pellets of the three complexes reveal clear emission bands for **Mo** and **W**, the maxima being localized at 666 and 673 nm (290 K), respectively (Figure 3). A weak but still detectable luminescence was observed for Cr around 700 nm. Hence, the spectral maxima follow the order **Cr**<**W**<**Mo**, as in the absorption spectra. The emission pattern of **Mo** shows a shoulder at 643 nm (290 K) beside the maximum at 666 nm, where the latter probably results from excitation of a low frequency vibration (∼500 cm^−1^) in the electronic ground state. At temperatures down to 5 K the emission maxima and shoulders shift to the blue by about 200 cm^−1^ for all three complexes (Figure 3). This may result from an inhibited excited state relaxation in the rigid matrix at very low temperature. Such effects are known in literature as rigidochromism^[36]^ and were observed for example for Re(I) carbonyl complexes before.^[37]^ Next, it is important to consider that the integrated emission intensity increases linearly by up to a factor of 4 upon cooling from 290 K to 5 K in the case of **Mo** (Figure S10, Supporting Information). An according increase stagnates below 100 K in the case of **W**, so that the total rise is limited in this case to a factor of 2.7. The higher luminescence at low temperature is explained by the inhibition of non‐radiative deactivation channels (e. g. vibrational relaxation), which arises as a consequence of an enhanced rigid environment. The quantum yields of the visible luminescence in the solid state at room temperature were determined to 1 ⋅ 10^−4^ and 2 ⋅ 10^−4^ for **Mo** and **W**, respectively (Table 1) (see Supporting Information for more details). These values are in the region of the quantum yields reported for other Mo(0) and W(0) carbonyl complexes.[^20^, ^37^] The VIS luminescence lifetimes in the solid state were determined to 8.1(1) ns (91 % contribution) and 2.04(6) ns (99 % contribution) by time‐correlated single photon counting (TCSPC) for **Mo** and **W** at 290 K, respectively (Table 1, Figures S12–S13, Tables S3–S4, Supporting Information). Upon cooling to 5 K the time constants increase to the respective values of 180(1) ns (88 % contribution) and 355(1) ns (81 % contribution), assigning the visible emission consequently to a triplet state (Figures S11, S14–S15, Tables S3–S4, Supporting Information). The shorter minor component (contribution of ≤ 19 %) might result from a second isomer or slightly different microenvironments and should not be explicitly assigned to a second VIS emissive excited state (see also discussion on static IR spectra, chapter 6 in the Supporting Information).

**Figure 3:**
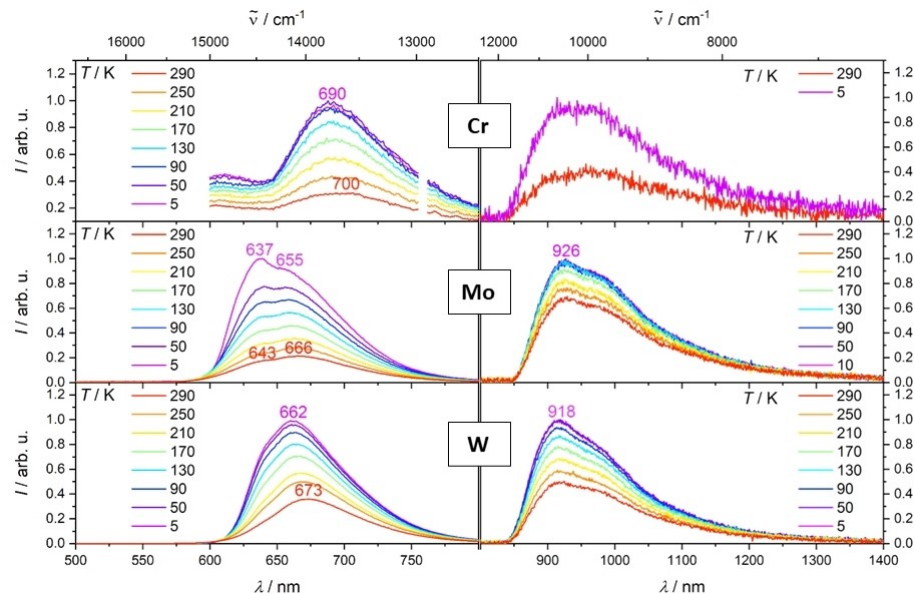
VIS (left) and NIR (right) emission spectra of **Cr**, **Mo** and **W** in the solid state (KBr pellets) at temperatures of 5–290 K measured at λ_ex_=420 nm.

**Table 1:** Emission maxima (λ_max_), luminescence quantum yields (*Φ*) and excited state lifetimes (τ ) of **Cr**, **Mo** and **W** in the solid state (KBr pellet) at 290 K.

Complex	λ_max_(VIS) [nm]	*Φ*(VIS)^[a]^	τ (VIS)^[b]^ [ns]	λ_max_(NIR) [nm]	*Φ*(NIR)^[a]^	τ (step‐scan) [ns]
Cr	≈695	–	–	≈965	–	380±10
Mo	666	1.1 ⋅ 10^−4^	8.1±0.1 (91 %)	926	9 ⋅ 10^−4^	600±10
W	673	1.7 ⋅ 10^−4^	2.04±0.06 (99 %)	918	1.4 ⋅ 10^−3^	370±10

[a] The error bars for the photoluminescence quantum yields are estimated to ±25 % according to the literature. [b] For the main component (relative contribution in brackets).^[38]^

Interestingly, all the three complexes show a second emission band, which reaches into the NIR II region. The **Cr** system shows a very weak luminescence around 950 nm, while the NIR luminescence is much stronger for the **Mo** and **W** derivatives with maxima at 926 nm and 918 nm, respectively (Figure 3). It should be highlighted that the broad emission reaches to 1350 nm, hence far into the NIR II region. The NIR and VIS emission spectra obtained with KBr pellets and neat powders of **Mo** are almost identical, so that any matrix effects are insignificant (Figure S17, Supporting Information). Furthermore, the very similar VIS and NIR excitation spectra confirm the assignment of both emission bands to one single species (Figure S18, see also discussion on the static IR spectra in the Supporting Information at the beginning of chapter 6). These are the first Cr(0), Mo(0) and W(0) complexes showing an NIR II emission, to the best of our knowledge. The quantum yield at 290 K even reaches very high values of 9 ⋅ 10^−4^ for **Mo** and 1.4 ⋅ 10^−3^ for **W** (Table 1). The emission maxima and the band‐shape with a shoulder around 1000 nm are not influenced by temperature, but the intensity increases by a factor of 1.3 and 1.8 upon cooling from 290 K to 100 K for **Mo** and **W**, respectively (Figure S16, Supporting Information). Similar to the VIS emission, the NIR luminescence intensity stagnates below 100 K.

Time‐resolved step‐scan FTIR spectroscopy was subsequently applied to analyze long‐lived electronically excited states and to obtain further information on the origin of the dual phosphorescence. The ground state FTIR spectra of **Cr**, **Mo** and **W** are very well described by the calculated S_0_ spectra (Figures 4, S26 and S29, Supporting Information). The solid samples (KBr pellets) were electronically excited at 532 nm and step‐scan difference spectra were recorded at 20 K and 290 K (Figures S20–S25, Supporting Information). The pure IR spectra of the long‐lived excited states were extracted by addition of a small contribution of the ground state spectrum to the corresponding step‐scan difference spectrum (see Supporting Information for more details). Interestingly, the excited state spectra at 20 K of **Mo** and **W**, averaged over the first 500 ns after laser excitation, strongly differ from the excited state absorption at longer time scales (>1 μs) (Figures 4 and S29, Supporting Information). At short time scales after the laser pulse, excited state specific vibrations are observed between 2000 and 1900 cm^−1^, which are very well described by a relaxed triplet state (labelled T_n_ in the following) optimized by unrestricted density functional theory (UDFT) (Figures 4 and S29, Supporting Information). The weak vibration predicted above 2050 cm^−1^ for both **Mo** and **W** is probably below the detection limit. The mentioned signals at 1900–2000 cm^−1^ decay monoexponentially with a sub‐microsecond lifetime (Figures S33 and S35, Table S5, Supporting) and are in good agreement with the luminescence lifetimes obtained by TCSPC (Tables 1, S3–S4, Supporting Information) for the VIS emission (see Supporting Information for the determination of excited state lifetimes by step‐scan FTIR).

**Figure 4:**
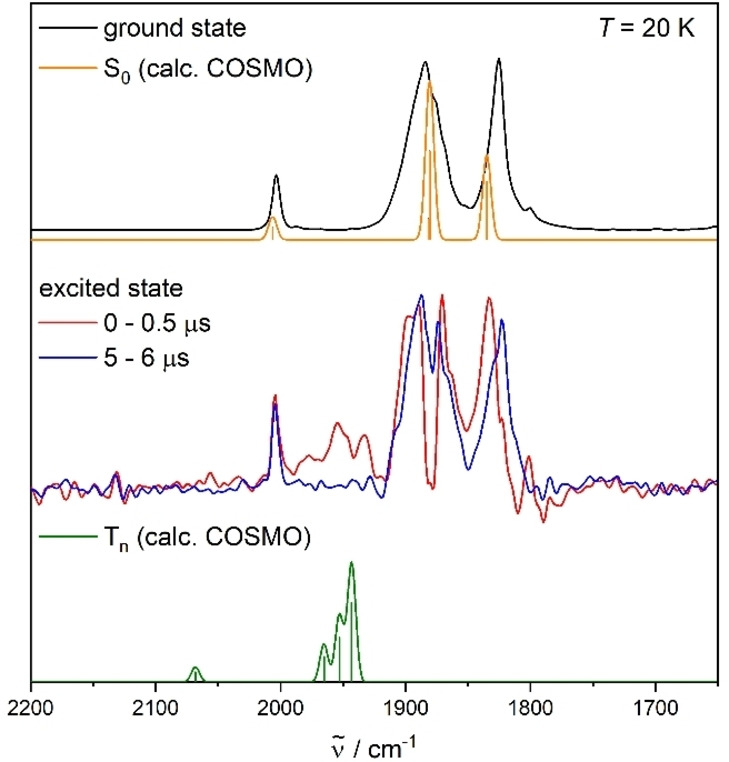
Ground state FTIR spectrum of **Mo** at 20 K (KBr pellet) and calculated S_0_ spectrum in KBr (top), excited state absorption spectra obtained from the step‐scan spectra at 0–0.5 μs and 5–6 μs after laser excitation (middle) as well as calculated triplet (T_n_) spectrum in KBr (bottom). The sticks represent the calculated IR absorption frequencies. Calculations: DFT/B3LYP‐D3(BJ)/def2‐TZVP/COSMO, IR absorption frequencies scaled by 0.975, convolution with Gaussian profile, FWHM=8 cm^−1^.

Hence, the mentioned short‐lived excited state IR absorption features are assigned to the triplet state with a VIS luminescence. This triplet state is of ^3^MLCT character with a charge transfer from the metal center to the bidentate pyridyl‐MIC ligand according to the calculated spin densities of these triplet states of **Mo** and **W** (Figures S40 and S41, Supporting Information). The calculated C−O bond lengths are slightly shorter in this triplet state than in the electronic ground state, while the M−CO bonds are elongated. These small structural changes can be explained by the weaker π ‐backbonding from the metal center to the CO ligands in the excited state (Figures S42–S44, Tables S9–S11, Supporting Information). The other step‐scan FTIR signals, apart from those assigned to the abovementioned triplet state (2000–1900 cm^−1^), are still observed at much longer time scales, and the deduced excited state spectra are similar to the IR absorption in the electronic ground state (Figures 4, S26–S30, Supporting Information). These excited state absorption features, which are close to the ground state IR spectrum, indicate that there are only small geometrical distortions with respect to the electronic ground state.

The mentioned transient IR signals show very long lifetimes above 100 μs at 20 K (Figures S31, S33, S35, Table S5, Supporting Information) strongly pointing towards a triplet state. This long‐lived triplet state is assigned to the observed NIR emission. The electronic character and structure of this NIR emissive excited state are currently investigated by further high‐level quantum chemical calculations. Analogous long‐lived transient IR signals were observed for **Cr**, apart from **Mo** and **W**, but no short‐lived excited state (Figures S26–S27, Supporting Information). The absence of these signals in the excited state IR spectrum may result from a small population of the underlying excited state, which would also explain the very weak VIS emission in this case. Simultaneously, the absence of these transient IR signals could result from a very short excited state lifetime (≤50 ns).

At 290 K the vibrations that are specific for the short‐lived excited state are hardly visible for **Mo** and completely absent for **W**, which probably results from the shorter excited state lifetimes at higher temperature (Figures S21, S23, S25, Supporting Information). Hence, the second excited state with a longer decay time almost exclusively contributes to the step‐scan spectrum, independent of the metal center. Even at room temperature the long‐lived triplet state still shows an excited state lifetime of several hundred nanoseconds (Table 1, Figures S32, S34, S36, Table S5, Supporting Information).

In summary, we presented Cr(0), Mo(0) and W(0) complexes with an NIR II emission. These systems contain the bidentate 1,2,3‐triazol‐5‐ylidene based mesoionic carbene ligand (PyC‐NMIC‐dipp) and CO coligands. The highest NIR quantum yield of 1.4 ⋅ 10^−3^ was achieved for W(0) in the solid state at room temperature. All the complexes show visible luminescence. The dual emission was assigned to two clearly separated phosphorescent triplet states (Figure 5) according to the lifetimes of at least hundreds of nanoseconds at low temperature, depending on the respective metal center and emission bands (VIS or NIR). The VIS emission was assigned to a ^3^MLCT state according to step‐scan FTIR spectroscopy and theoretical calculations, while the character of the NIR luminescent triplet state is currently analyzed by further high‐level quantum chemical calculations. Related dual emissive M(CO)_4_L complexes (M=Cr, Mo, W and L=*N*,*N*‐donor ligand) reported in the literature[^18^, ^37^] and the presented TDDFT calculations with several ^1^MLCT excitations indicate that also the NIR emissive triplet state might be of ^3^MLCT character. Furthermore, the large energy separation of about 5400 cm^−1^ between the two lowest energy ^1^MLCT excitations could in analogy explain the large energy separation between two emissive ^3^MLCT states (comparable S‐T gaps).

**Figure 5:**
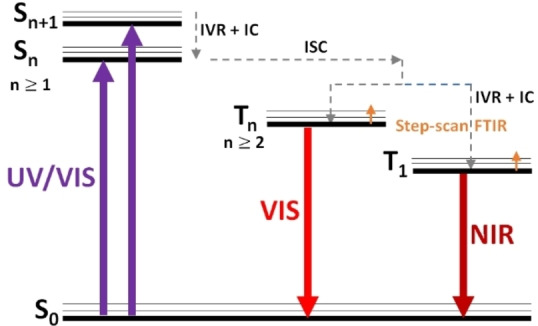
Simplified Jablonski diagram summarizing the photophysics of Cr, Mo and W. (IVR: intravibrational relaxation, IC: internal conversion, ISC: intersystem crossing)

This work represents an important milestone for the design and synthesis of further efficient NIR emitters based on earth‐abundant metals.

## Conflict of interest

The authors declare no conflict of interest.

## Supporting information

As a service to our authors and readers, this journal provides supporting information supplied by the authors. Such materials are peer reviewed and may be re‐organized for online delivery, but are not copy‐edited or typeset. Technical support issues arising from supporting information (other than missing files) should be addressed to the authors.

Supporting Information
