# Supplementary material for: NIR‐Emissive Chromium(0), Molybdenum(0), and Tungsten(0) Complexes in the Solid State at Room Temperature
Source: Chemistry. 2021 Aug 4;27(51):12959–64. doi: 10.1002/chem.202102208 (PMC8519045; doi:10.1002/chem.202102208)
Supplement: Supplementary file 1 — Supporting Information [file CHEM-27-12959-s001.pdf]

Chemistry–A European Journal

Supporting Information

NIR-Emissive Chromium(0), Molybdenum(0), and Tungsten(0) Complexes in the Solid State at Room Temperature

Pit Boden, Patrick Di Martino-Fumo, Tobias Bens, Sophie Steiger, Uta Albold, Gereon Niedner-Schatteburg, Markus Gerhards[+],* and Biprajit Sarkar*

Supporting Information

Table of contents

1. Experimental details 2
2. Theoretical Methods 4
3. Additional data on W 5
4. Experimental and calculated UV/VIS absorption spectra 11
5. Luminescence data 16
6. Static and transient step-scan FTIR spectroscopy 22
7. Additional calculated data 37
8. References 51

1. Experimental details

Preparation of KBr pellets

KBr pellets were prepared by mixing neat powder of the compound (0.4 mg − 1.8 mg, depending on the complex and the experiment) with dry KBr (200 – 300 mg, stored in a compartment dryer at 80 °C, purchased from Merck) and grinding to a homogenous mixture. This mixture was filled in an evacuable pellet die with a diameter of 13 mm and sintered at a pressure of 0.75 GPa. Please consider the respective sections for more details on the specific sample preparations.

UV/VIS absorption spectroscopy

UV/VIS absorption experiments in solution were performed with a Lambda 900 UV/VIS spectrometer in different solvents (CH_3CN, CH_2Cl_2 and toluene) using 10 mm path length quartz cells at 25°C. The solutions were prepared using the common Schlenk technique with concentrations in the range of $2 \cdot 10^{-5}$ M. The spectra were recorded relative to the pure solvent.

UV/VIS reflectance spectra were recorded with the same spectrometer by incorporation of a diffuse reflectance accessory (Harrick Praying Mantis) into the sample compartment. The KBr pellets were prepared by applying the procedure described above with 1.4 mg, 1.5 mg and 1.8 mg of sample for **Cr**, **Mo** and **W**, respectively, as well as 300 mg of KBr.

Temperature-dependent static UV/VIS emission spectroscopy

Temperature-dependent steady state luminescence measurements in the UV/VIS region were performed using a Horiba Jobin Yvon Fluorolog 3-22 τ spectrometer equipped with a 450 W xenon lamp and a R928 P photomultiplier detector (240 nm $< \lambda_{\mathrm{em}} <$ 850 nm). Spectral selection was achieved with double grating monochromators in the excitation and emission paths with 1200 grooves/mm (300 nm and 500 nm blaze in the excitation and emission paths, respectively). All the presented emission spectra are shown as obtained from the response of the instrument. Calibration of the excitation monochromator was performed according to the peak at 467 nm in the emission spectrum of the used xenon lamp. The UV/VIS and NIR emission monochromators were calibrated according to a water Raman spectrum and the 1064 nm peak of a Nd:YAG rod, respectively. Long-pass filters (cut-on wavelength of 600 nm for **Cr**, 540 nm for **Mo** and 500 nm for **W**) were used in the emission channel to avoid higher order excitation light. KBr pellets were prepared as described in the section on the preparation of KBr pellets using 1.5 mg, 1.0 mg and 1.2 mg of sample for **Cr**, **Mo** and **W**, respectively, as well as 200 mg of KBr. Measurements with neat powders of **Mo** were performed by homogenous spreading of the neat sample between two CaF_2 windows (13 mm diameter, 1 mm thick). Temperature-dependent experiments between 5 K and 290 K were performed using a closed-cycle helium cryostat (ColdEdge, 101J cryocooler) to cool down the sample. The cryocooler was equipped with a pellet holder (copper) and CaF_2 windows.

Temperature-dependent static NIR emission spectroscopy

Temperature-dependent steady state NIR luminescence measurements were conducted on a Horiba Jobin Yvon Fluorolog 3-22 τ spectrometer equipped with a 450 W xenon lamp and a DSS – IGA020L NIR detector (850 nm $\leq \lambda_{\mathrm{em}} \leq$ 1550 nm). Spectral selection was realized with double and single grating monochromators in the excitation and emission paths, respectively (excitation: 1200 grooves/mm; near-IR emission 600 grooves/mm). A combination of two long-pass filters (FELH0500 Thorlabs, transmission ≥ 92% above 500 nm and FELH0850 Thorlabs, transmission ≥ 90% above 860 nm) was used in the emission channel to avoid higher order excitation light.

KBr pellets and powders were prepared as described for the UV/VIS emission spectroscopy. Experiments on **Cr** and **W** at temperatures between 5 K and 290 K were performed using the

cryostat described above. Measurements on **Mo** were conducted at temperatures down to about 10 K by using an analogous closed-cycle helium cryostat (ARS Model DE-202A).

Temperature-dependent time-correlated single photon counting (TCSPC) for UV/VIS luminescence

UV/VIS luminescence lifetimes were determined by time-correlated single photon counting (TCSPC) using a DeltaFlex (Horiba Scientific) spectrometer. The sample was excited with short light pulses of a NanoLED 390 (peak wavelength: 389 nm, pulse duration: 1.3 ns). A long-pass filter (cut-on wavelength of 600 nm) was placed in the emission channel to suppress the influence of scattered excitation light. The emission monochromator was set to the respective emission band of the investigated compound. Precision photon counting was carried out with a PPT (picosecond photon counting) detection module, including a fast-rise photomultiplier with an integral GHz timing preamplifier, a constant fraction discriminator and a regulated HV supply. Decay curves were analyzed by multiexponential fits with the software ORIGIN®. KBr pellets were prepared as described for the static luminescence measurements. The low-temperature measurements were performed using the cryostat presented in the section on static UV/VIS emission spectroscopy.

Determination of luminescence quantum yields

Absolute UV/VIS photoluminescence quantum yields of solid samples (KBr pellets) were measured in analogy to the procedures described by Wrighton et al. and latter Liu et al., who measured fluorescence quantum yields with a conventional fluorescence spectrometer.[1,2]
All spectra were recorded at room temperature on the Horiba Jobin Yvon Fluorolog 3-22 τ spectrometer described above with the KBr pellets being mounted in a solid state sample holder. The pellets were prepared as described for solid state UV/VIS absorption spectroscopy.
The absorption of the complex was measured by considering the difference in area between the excitation light (λ_{ex} = 380 nm) scattered by a neat KBr pellet (J_0) and a pellet containing the complex (J). For the luminescence the area J_f under the emission curve was considered. The photoluminescence quantum yield ϕ was then calculated according to the following formula:

$$\phi_{VIS} = \frac{J_f}{J_0 - J} \quad (1)$$

We tested the procedure on our setup by determination of the literature known quantum yield of the luminescent copper complex [(2-(Diphenylphosphino)pyridine)(tris(4-fluorophenyl)phosphine)$_2$Cu$_2$I$_2$] to 70%, in accordance with the literature-known values of 59 – 74% in different host matrices (10wt%).[3]

Similar methods were applied for the determination of NIR photoluminescence quantum yields. The main difference compared to the procedures described above for the VIS emission is that the quantum yields were measured relative to the standard Yb(tta)$_3$(H$_2$O)$_2$ with $\phi_r(Yb)$ = 0.55% as PMMA film.[4] This relative determination was applied as the scattered excitation (λ_{ex} = 350 nm) and NIR emission light could not be recorded with the same detector (see the sections on static luminescence spectroscopy for more details on the used spectrometer Fluorolog 3-22 τ). The NIR photoluminescence quantum yield was calculated according to the following equation, where the abbreviation S stands for the investigated sample with unknown quantum yield and Yb represents the reference Yb(tta)$_3$(H$_2$O)$_2$:

$$\phi_{NIR} = \frac{J_0 - J(Yb)}{J_0 - J(S)} \cdot \frac{J_f(S)}{J_f(Yb)} \cdot \phi_r(Yb) \quad (2)$$

The error bars for the photoluminescence quantum yields are estimated to ± 25% for the applied procedures according to literature.[1]

Time-resolved step-scan FTIR spectroscopy

All the time-resolved FTIR experiments were performed with the FTIR spectrometer Bruker Vertex 80v, operated in the step-scan mode. A liquid-nitrogen-cooled mercury cadmium telluride (MCT) detector (Kolmar Tech., Model KV100-1-B-7/190) with a rise time of 25 ns, connected to a fast preamplifier and a 14-bit transient recorder board (Spectrum Germany, M3I4142, 400 MS/s), was used for signal detection and processing. The laser setup used for the measurements includes a Q-switched Nd:YAG laser (Innolas SpitLight Evo I) generating pulses with a duration of about 6 ns at a repetition rate of 100 Hz. The second harmonic (532 nm) of the Nd:YAG laser was used directly for sample excitation. The UV pump beam was attenuated to about 2.0 mJ per shot at a diameter of 9 mm. The beam was directed onto the sample and adjusted to have a maximal overlap with the IR beam of the spectrometer. The sample chamber was equipped with anti-reflection-coated germanium filters to prevent the entrance of laser radiation into the detector and interferometer compartments.

The KBr pellets were prepared as described in the section on luminescence spectroscopy, however, with a smaller amount of sample of ca. 0.4 mg and ca. 200 mg KBr. The strongest peak in the ground state spectrum showed an absorption of about 0.6 OD with the mentioned concentration. The measurements were performed at a constant temperature of 290 K or 20 K using a closed-cycle helium cryostat (ARS Model DE-202A). The cryocooler was equipped with a pellet holder and CaF_2 windows.

The temporal resolution of the 14-bit transient recorder board was chosen to 50 ns and 10000 time slices were recorded to cover a time range of 500 µs. The time when the laser pulse reached the sample was set as zero point in all spectra. The time delay between the start of the experiment and the laser pulse was controlled with a Stanford Research Systems DG535 delay generator and set to 1.3 µs before the laser excitation of the sample. The spectral region was limited by undersampling to 2634 – 0 cm^{-1} with a spectral resolution of 4 cm^{-1} resulting in 1481 interferogram points. An IR long pass filter (no IR transmission < 2400 cm^{-1}) prevented problems when performing a Fourier transformation (i.e. no IR intensity outside the measured region should be observed). FTIR ground state spectra were recorded systematically to check if there is no sample degradation.

2. Theoretical Methods

The crystal structures were used as input structures and geometry optimizations were performed with the Berny algorithm of Gaussian 09[5] by using energies and gradients computed by Turbomole 7.4.[6,7] All calculations were performed with the DFT functional B3LYP with dispersion correction (no three-body interaction) (D3(BJ))[8] as implemented in Turbomole using the resolution of identity (RI) approximation and the def2-TZVP basis set.
Turbomole 7.4[6,7] was used for computing the first hundred electronic excitations in the singlet manifold with TDDFT and simulate the UV/VIS spectra using the same functional and basis set as described above. For convolution Gaussian broadening with a full-width at half maximum of 1500 cm^{-1} was used. The influence of the medium (CH_3CN, CH_2Cl_2, KBr) was modulated by using the conductor-like screening model (COSMO).
Harmonic frequency calculations were performed for the optimized minimum structures. The influence of the KBr matrix was modulated by using the conductor-like screening model (COSMO). The vibrational frequencies are scaled by a factor of 0.975 to minimize the differences between the experimental and calculated frequencies. A gaussian convolution with a full-width at half-maximum of 8 cm^{-1} was applied to the calculated vibrational transitions.

3. Additional data on W

General Procedures, Materials and Instrumentation

Caution! *Compounds containing azides are potentially explosive. Although we never experienced any problems during synthesis or analysis, all compounds should be synthesized only in small quantities and handled with great care!*

Unless otherwise noted, all reactions were carried out using standard Schlenk-line-techniques under an inert atmosphere of argon (Linde Argon 4.8, purity 99.998%).

Commercially available chemicals were used without further purification. The solvents used for metal complex synthesis and catalysis were available from Glovebox Systemtechnik solvent System and degassed by standard techniques prior to use. The identity and purity of compounds were established *via* ^{1}H NMR and IR spectroscopy, elemental analysis and mass spectrometry.

Column chromatography was performed over silica 60 M (0.04 − 0.063 mm).

UV-Irradiation for synthesis was performed with a LSN150/2 (LOT Quantum Design GmbH) at 150 W.

^{1}H and $^{13}C\{^{1}H\}$ NMR spectra were recorded on a Bruker Avance 700 spectrometer at 19 – 22 °C. Chemical shifts are reported in ppm referenced to the residual solvent peaks.[9]

The following abbreviations are used to represent the multiplicity of the signals: s (singlet), d (doublet), t (triplet), q (quartet), p (pentet), sept (septet).

Mass spectrometry was performed on an Agilent 6210 ESI-TOF.

The IR spectra were recorded with a BRUKER Vertex 70 FT-IR spectrometer.

Elemental analyses were performed with an Elementar Micro Cube elemental analyser.

X-ray Diffraction

X-ray data were collected on a BRUKER D8 Venture system. Data were collected at 103(2) K, using graphite-monochromated Mo K_α radiation (λ_α = 0.71073 Å). The strategy for the data collection was evaluated by using the APEX2 or Smart software. The data were collected by standard "ω scan techniques" or "$\omega - \varphi$ scan techniques" and were scaled and reduced using APEX2, SAINT+, and SADABS software. The structures were solved by direct methods using SHELXL-97 or intrinsic phasing using SHELXL-2014/7 and refined by full matrix least-squares with SHELXL-2014/7, refining on F^2. Non-hydrogen atoms were refined anisotropically.

Preparation of W

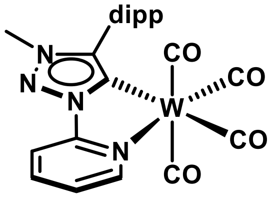

A suspension of [$W(CO)_6$] (45 mg, 0.128 mmol) in 20 mL CH_3CN was stirred for 2 hours under UV light. The CO overpressure was released at least 3 times during this period. **[HL1]OTf** (60 mg, 0.128 mmol) and an excess of NEt_3 (1.0 mL) were added. The mixture was refluxed with an equipped reflux condenser and a gas bubbler for 3 days. After cooling to room temperature, the solvent was evaporated and the remaining residue was dissolved in CH_2Cl_2 and extracted two times with H_2O. The organic phases were collected and dried over Na_2SO_4. The solvent was removed and the crude product was purified by column chromatography (SiO_2, 100% CH_2Cl_2). Analytically pure product was isolated after recrystallization from CH_2Cl_2 and *n*-hexane yielding red crystals (47 mg, 60%).

^{1}H NMR (700 MHz, $CDCl_3$) δ (ppm) = 9.15 (dq, *J* = 5.6 Hz, 0.8 Hz, 1H), 8.19 (d, *J* = 8.3 Hz, 1H), 8.04 (td, *J* = 7.9 Hz, 1.6 Hz, 1H), 7.55 (t, *J* = 7.9 Hz, 1H), 7.37-7.30 (m, 3H), 3.93 (s, N-C<u>*H*</u>$_3$, 3H), 2.60 (sept, *J* = 6.8 Hz, dipp-<u>*H*</u>, 2H), 1.30 (d, *J* = 6.8 Hz, dipp-C<u>*H*</u>$_3$, 6H), 1.14 (d, *J* = 6.8 Hz, dipp-C<u>*H*</u>$_3$, 6H); **MS (ESI)**: m/z found: 588.1370, calcd: 588.1358 [$C_{23}H_{24}N_4O_3W^+$], 611.1233, calcd: 611.1250 [$C_{23}H_{24}N_4NaO_3W^+$], 627.0969, calcd: 627.09689 [$C_{23}H_{24}N_4KO_3W^+$], 321.2074, cacld: 321.2079 [$C_{20}H_{25}N_4^+$]; **IR** ($\tilde{\upsilon}$(CO), CH_3CN) = 2000 cm^{-1} (s), 1882 cm^{-1} (s), 1870 cm^{-1} (sh), 1827 cm^{-1} (s); **Anal. calcd. for $C_{24}H_{24}WN_4O_4$**: C, 46.77, H, 3.93, N, 9.09; **found**: C, 46.66, H, 3.94, N, 9.00.

NMR Spectrum of W

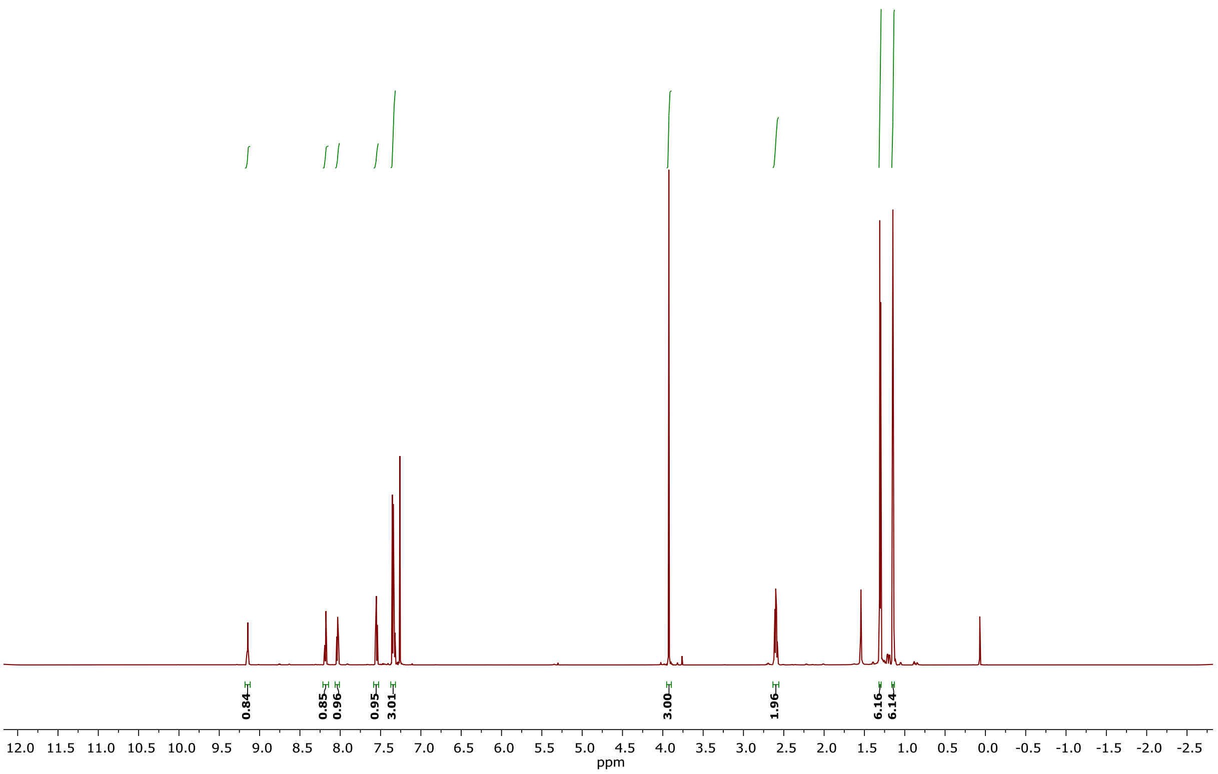

Figure S1. ^{1}H NMR (700 MHz, $CDCl_3$) spectrum of **W**.

IR Spectrum of W

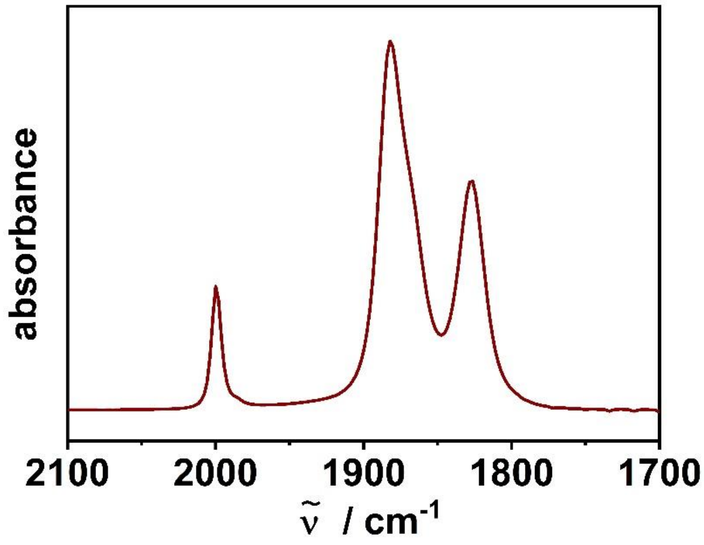

Figure S2. IR spectrum of **W** in CH_3CN.

Single Crystal X-Ray Diffraction Data & Crystal Structure

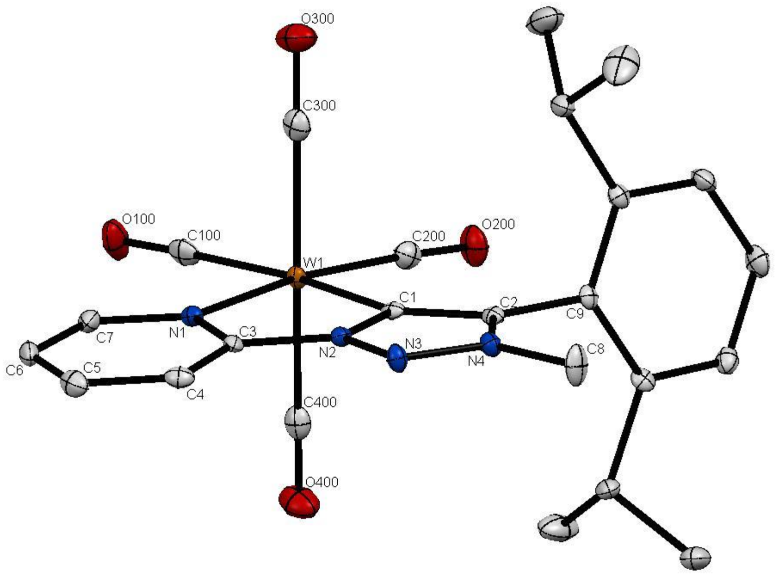

Figure S3. ORTEP representation of **W** (hydrogen atoms are omitted for clarity). Ellipsoids are drawn with 50% probability.

Accession Codes

CCDC 2064469 contain the supplementary crystallographic data for this paper. These data can be obtained free of charge viawww.ccdc.ca-m.ac.uk/data_request/cif, or by emailingdata_request@ccdc.cam.ac.uk, or by contacting The Cambridge Crystallographic Data Centre, 12 Union Road, Cambridge CB2 1EZ, UK; fax: +44 1223 336033.

Table S1. Selected bond lengths and angles of **W**.

**Atoms**	**W**
	**Bond lengths / Å**
W1-C1	2.186(3)
W1-N1	2.259(2)
W1-C100	1.990(3)
W1-C200	1.974(3)
W1-C300	2.039(3)
W1-C400	2.028(3)
C100-O100	1.164(4)
C200-O200	1.160(4)
C300-O300	1.150(4)
C400-O400	1.155(4)
C1-C2	1.398(4)
C2-N4	1.366(4)
N4-N3	1.327(3)
N3-N2	1.349(3)
C1-N2	1.374(4)
N2-C3	1.414(3)
C3-N1	1.341(4)
N1-C7	1.357(4)
C7-C6	1.367(4)
C6-C5	1.393(4)
C5-C4	1.384(4)
C4-C3	1.381(4)
	**Bond angles / °**
C400-W1-C300	171.2(1)
C100-W1-C1	167.2(1)
C200-W1-N1	169.7(1)
N1-W1-C1	72.7(1)
C2-C9	76.1(1)

Table S2. Crystollographic data for **W**

	**W**
Chemical formula	$C_{48}H_{48}N_8O_8W_2$
M_r	1232.64
Crystal system	Monoclinic
Space group	*P*2(1)/*c*
a (Å)	11.8022(9)
b (Å)	16.0693(13)
c (Å)	13.1197(10)
α (°)	90
β (°)	111.641(3)
γ (°)	90
V (Å^3)	2312.8(3)
Z	2
Densitiy (g cm^{-3})	1.770
F(000)	1208
Radiation Type	MoK_α
μ (mm^{-1})	5.033
Crystal size	0.38 x 0.33 x 0.2
Meas. Refl.	49464
Indep. Refl.	6995
Obsvd. [$I > 2\sigma(I)$] refl.	5638
R_{int}	0.0629
R [$F^2 > 2\sigma(F^2)$], wR(F^2), S	0.0294, 0.0545, 0.995
$\Delta\rho_{max}$, $\Delta\rho_{min}$ (e Å^{-3})	0.679, −1.153

4. Experimental and calculated UV/VIS absorption spectra

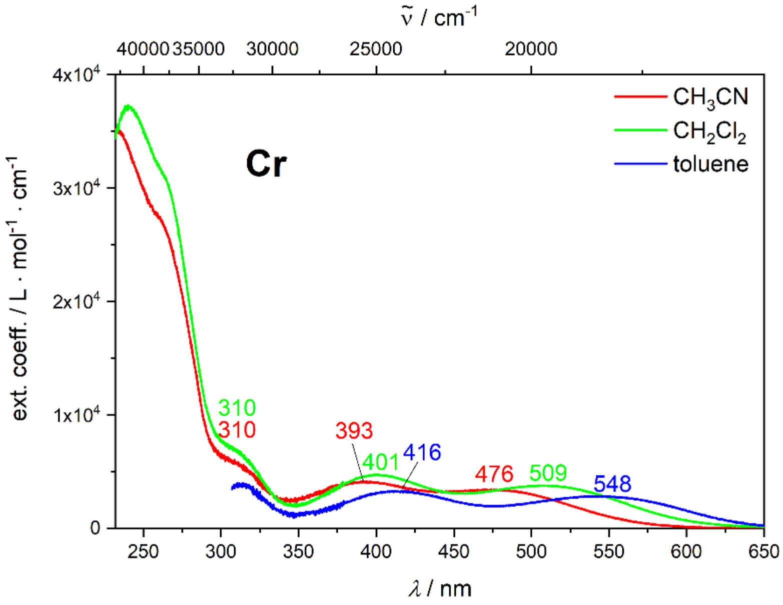

Figure S4. UV/VIS absorption spectra of **Cr** in CH_3CN, CH_2Cl_2 and toluene.

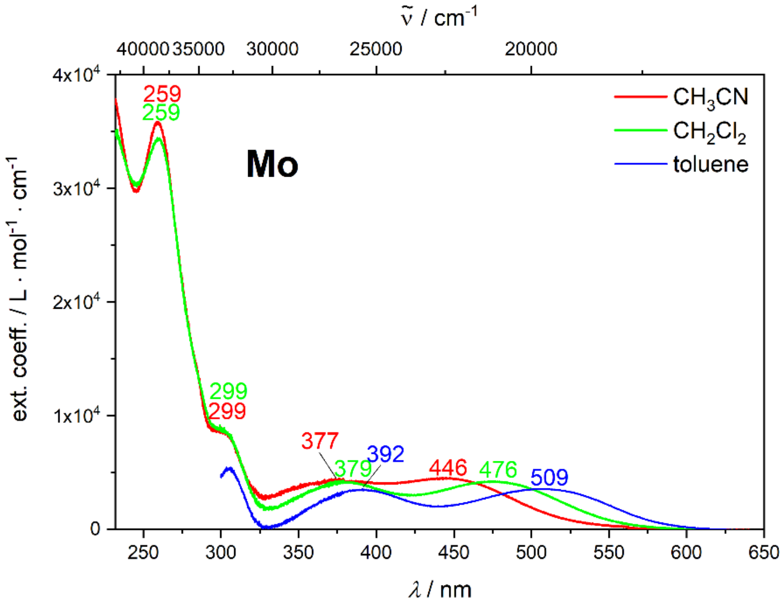

Figure S5. UV/VIS absorption spectra of **Mo** in CH_3CN, CH_2Cl_2 and toluene.

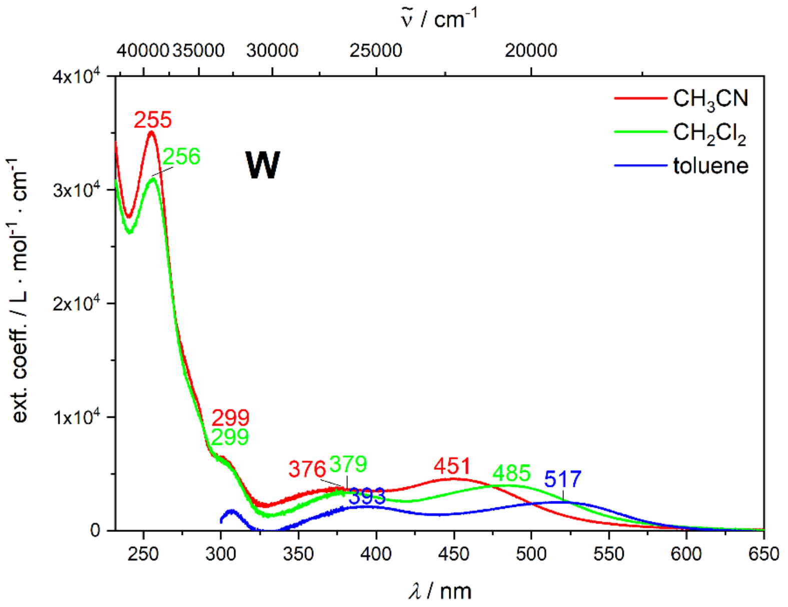

Figure S6. UV/VIS absorption spectra of **W** in CH_3CN, CH_2Cl_2 and toluene.

Comparison with calculated UV/VIS absorption spectra

The pronounced solvatochromism of the low energy absorption bands of **Cr**, **Mo** and **W** was confirmed by TDDFT calculations in CH_3CN and CH_2Cl_2. Additionally, calculations were performed in KBr. The calculated spectra of **Cr** and **Mo** are in very good agreement with the experiment. The larger deviations between the measured and theoretical spectra in the case of **W** probably result from a less accurate description by using an effective core potential for tungsten as implemented in turbomole.

The UV/VIS absorption spectra of **Cr**, **Mo** and **W** have very similar patterns and also similar absorption maxima. Furthermore, the character of the underlying electronic transitions is not significantly affected by the metal center and the medium according to theory. Hence, the assignment of these transitions is given exemplary in the last chapter for **Mo** in CH_3CN, CH_2Cl_2 and KBr. At this point it should also be mentioned that the medium has only a very small impact on the shape of the molecular orbitals, which are also presented in the last section.

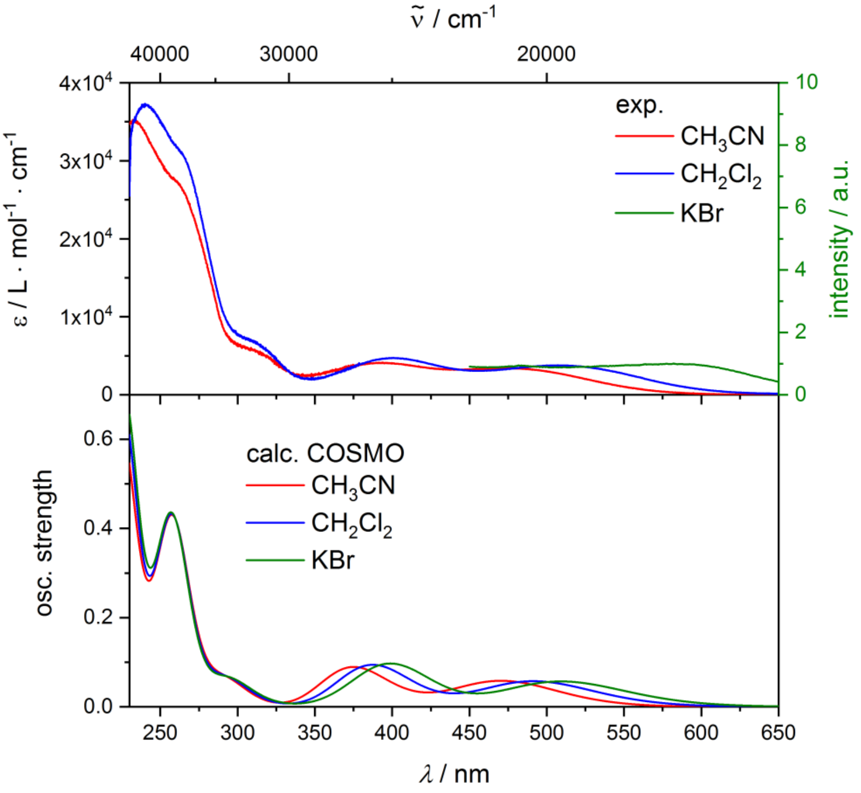

Figure S7. Experimental UV/VIS absorption spectra of **Cr** in CH_3CN, CH_2Cl_2 and as KBr pellet (top). UV/VIS absorption spectra were calculated in CH_3CN, CH_2Cl_2 and KBr for comparison (bottom). Calculations: TDDFT/B3LYP-D3(BJ)/def2-TZVP/COSMO.

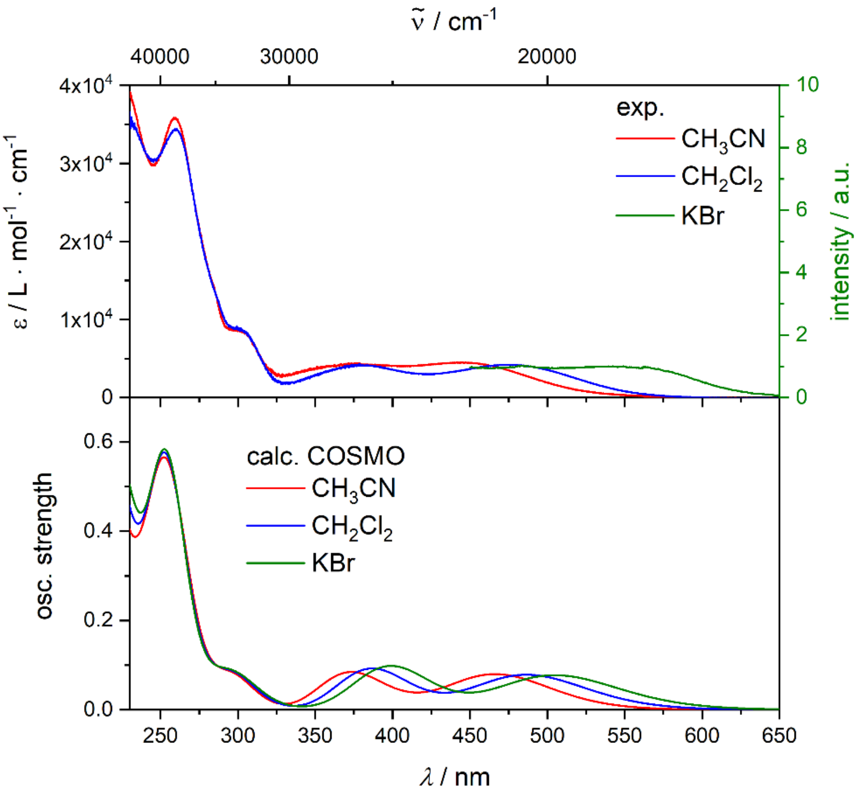

Figure S8. Experimental UV/VIS absorption spectra of **Mo** in CH_3CN, CH_2Cl_2 and as KBr pellet (top). UV/VIS absorption spectra were calculated in CH_3CN, CH_2Cl_2 and KBr for comparison (bottom). Calculations: TDDFT/B3LYP-D3(BJ)/def2-TZVP/COSMO.

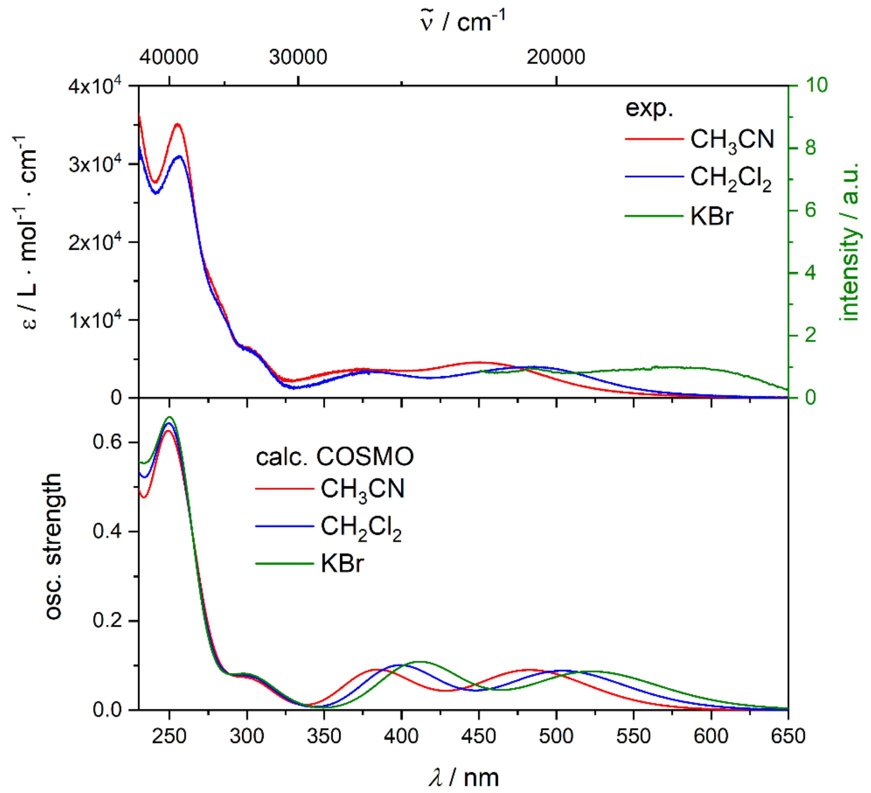

Figure S9. Experimental UV/VIS absorption spectra of **W** in CH_3CN, CH_2Cl_2 and as KBr pellet (top). UV/VIS absoprtion spectra were calculated in CH_3CN, CH_2Cl_2 and KBr for comparison (bottom). Calculations: TDDFT/B3LYP-D3(BJ)/def2-TZVP/COSMO.

5. Luminescence data

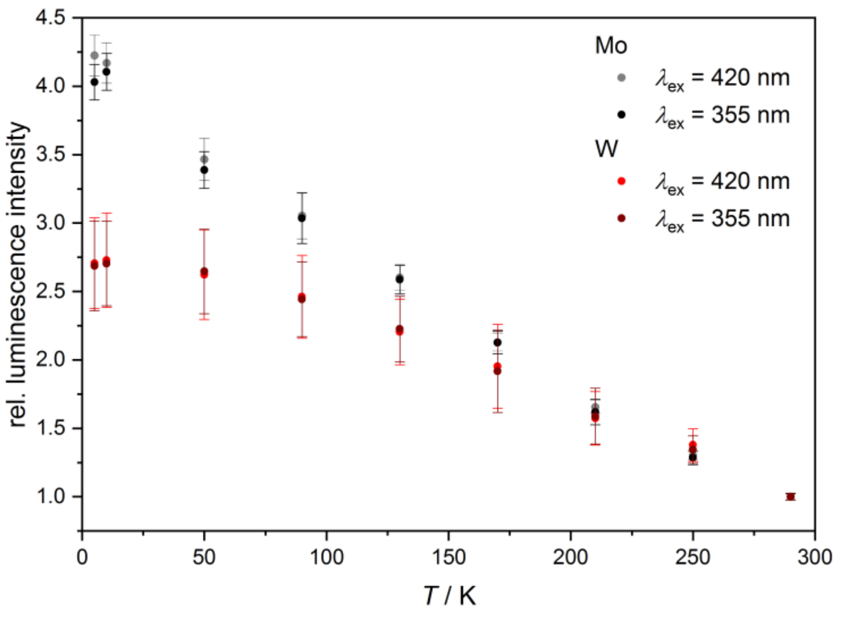

Figure S10. Integrated visible luminescence intensity (spectral region of 575 – 800 nm) relative to 290 K for **Mo** and **W** (KBr pellet, T = 290 – 5 K, λ_{ex} = 355, 420 nm).

Table S3. Temperature-dependent luminescence lifetimes of **Mo** measured by time-correlated single photon counting (TCSPC, KBr pellet, λ_{ex} = 345 nm).

**Complex**		**Mo**			
***T* / K**	**λ_{em} / nm**	**t_1 / ns**	**A_1 /%**	**t_2 / ns**	**A_2 /%**
**290**	666	**8.1** ± 0.1	91	24.0 ± 0.4	9
	643	**7.8** ± 1	89	29.0 ± 0.5	11
**210**	664	6.4 ± 0.2	14	**21.1** ± 0.1	86
	641	7.0 ± 0.1	21	**23.7** ± 0.2	79
**90**	659	6.3 ± 0.1	4	**55.8** ± 0.1	96
	641	6.6 ± 0.1	9	**56.2** ± 0.2	91
**50**	657	13.6 ± 0.5	16	**79.2** ± 0.4	84
	640	7.6 ± 0.2	18	**74.2** ± 0.3	82
**25**	655	22 ± 1	18	**100** ± 1	82
	637	19.8 ± 0.5	22	**102** ± 1	78
**5**	655	24.7 ± 0.4	7	**170** ± 1	93
	637	22.3 ± 0.3	12	**180** ± 1	88

Table S4. Temperature-dependent luminescence lifetimes of **W** measured by time-correlated single photon counting (TCSPC, KBr pellet, λ_{ex} = 345 nm).

**Complex**		**W**			
***T* / K**	**λ_{em} / nm**	**t_1 / ns**	**A_1 /%**	**t_2 / ns**	**A_2 /%**
**290**	673	**2.04** ± 0.06	99	10.8 ± 0.1	1
**210**	668	3.4 ± 0.1	9	**17.5** ± 0.1	91
**90**	663	7.1 ± 0.2	4	**42.0** ± 0.1	96
**50**	662	9.1 ± 0.2	15	**73.0** ± 0.2	85
**25**	662	40 ± 1	14	**160** ± 1	86
**5**	662	18.1 ± 0.3	19	**355** ± 1	81

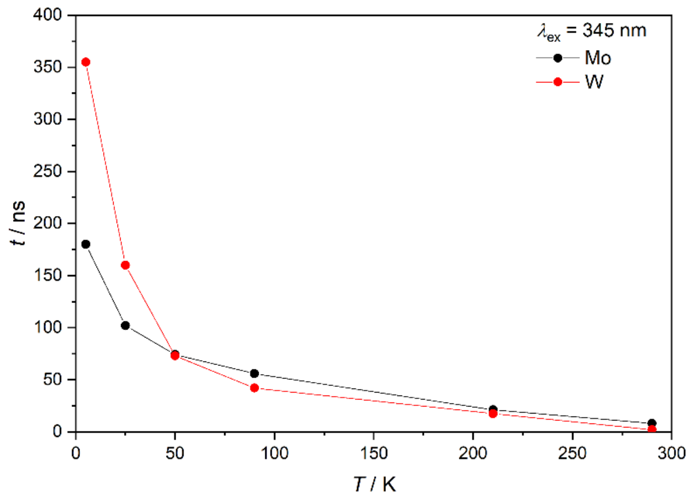

Figure S11. Plot of the luminescence lifetime of **Mo** and **W** against temperature (dominating time constant t_2, see tables S3 and S4 for **Mo** and **W**, respectively). Error bars were deduced from the fit, but are smaller than the dots.

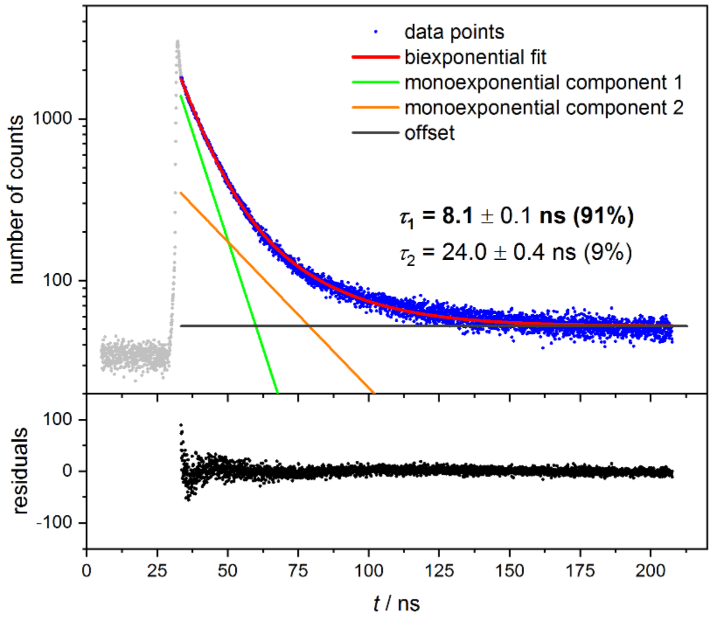

Figure S12. Measured luminescence decay curve (blue dots) of the visible emission band of **Mo** at 290 K (λ_{ex} = 345 nm; λ_{em} = 666 nm). The red curve shows the biexponential fit (including an offset) and the green, orange and grey lines represent the underlying monoexponential components and the offset, respectively. The grey dots represent the excitation pulse, which is not considered in the fit. The lower trace shows the residuals of the fit.

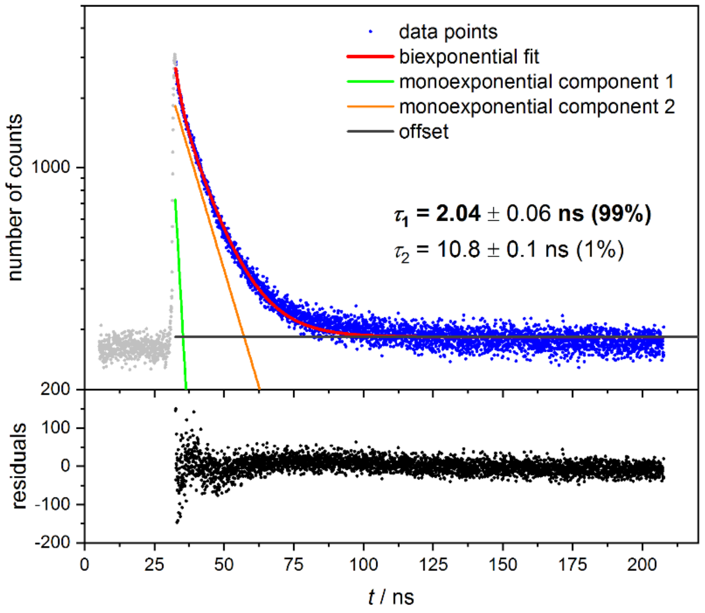

Figure S13. Measured luminescence decay curve (blue dots) of the visible emission band of **W** at 290 K (λ_{ex} = 345 nm; λ_{em} = 673 nm). The red curve shows the biexponential fit (including an offset) and the green, orange and grey lines represent the underlying monoexponential components and the offset, respectively. The grey dots represent the excitation pulse, which is not considered in the fit. The lower trace shows the residuals of the fit.

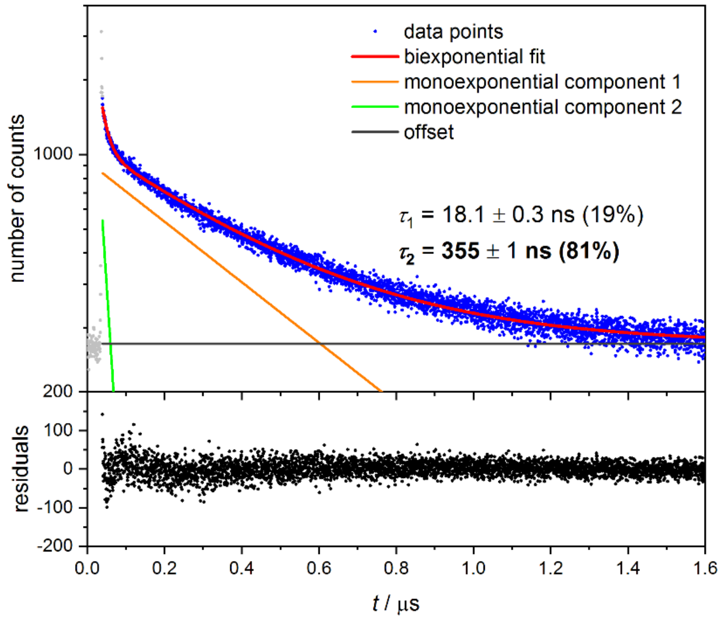

Figure S14. Measured luminescence decay curve (blue dots) of the visible emission band of **Mo** at 5 K (λ_{ex} = 345 nm; λ_{em} = 655 nm). The red curve shows the biexponential fit (including an offset) and the green, orange and grey lines represent the underlying monoexponential components and the offset, respectively. The grey dots represent the excitation pulse, which is not considered in the fit. The lower trace shows the residuals of the fit.

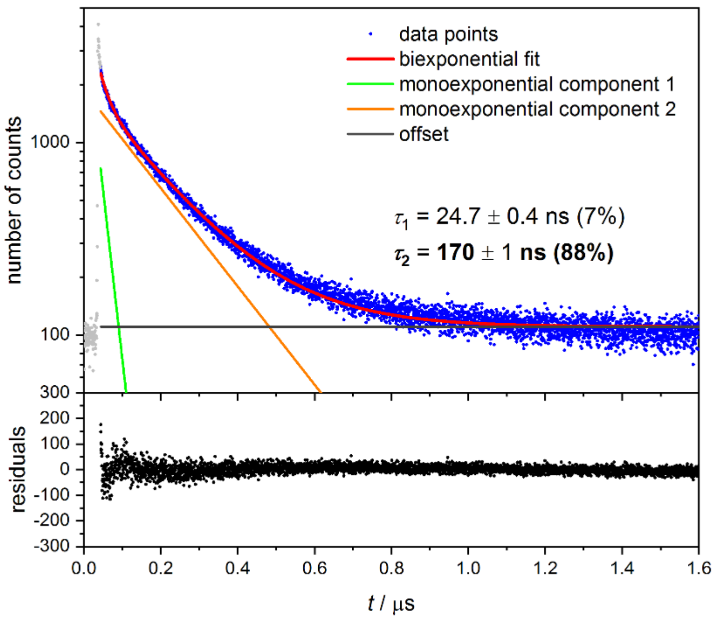

Figure S15. Measured luminescence decay curve (blue dots) of the visible emission band of **W** at 5 K (λ_{ex} = 345 nm; λ_{em} = 662 nm). The red curve shows the biexponential fit (including an offset) and the green, orange and grey lines represent the underlying monoexponential components and the offset, respectively. The grey dots represent the excitation pulse, which is not considered in the fit. The lower trace shows the residuals of the fit.

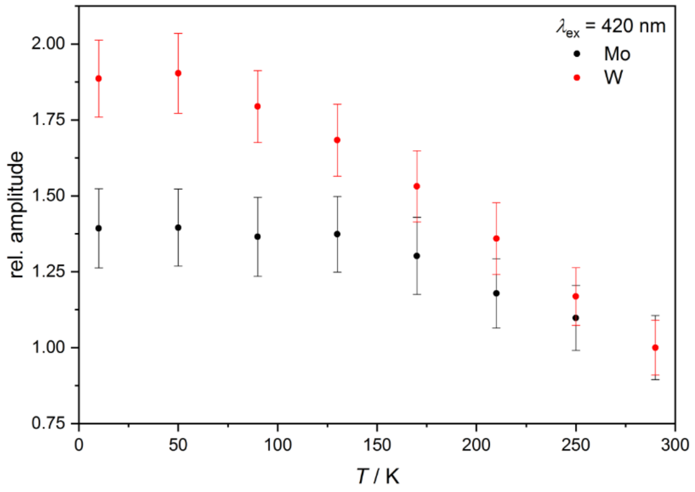

Figure S16. Integrated NIR luminescence intensity (spectral region of 800 — 1400 nm) relative to 290 K for **Mo** and **W** (KBr pellet, T = 290 — 5 K, λ_{ex} = 420 nm).

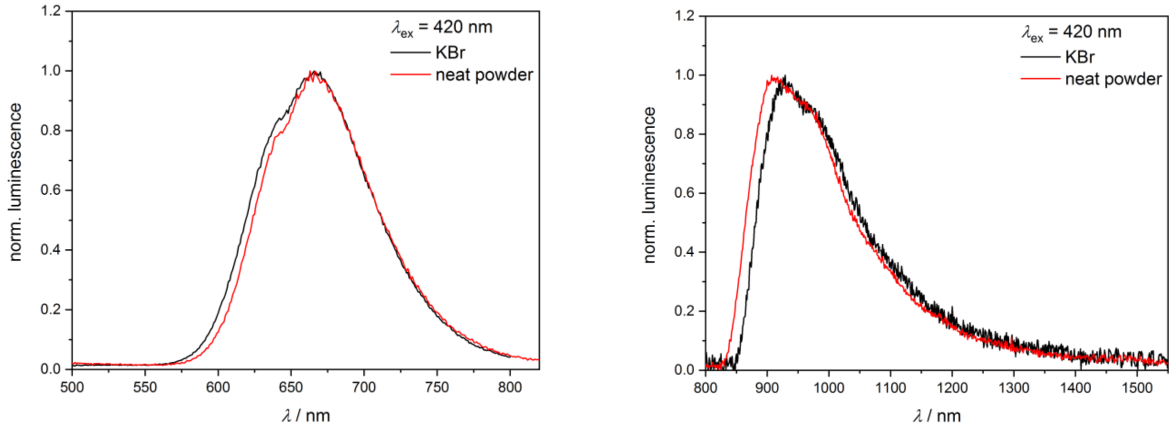

Figure S17. Emission spectra of **Mo** in the VIS (left) and NIR (right) regions, measured as KBr pellet (black) and neat powder (red) at room temperature (λ_{ex} = 420 nm).

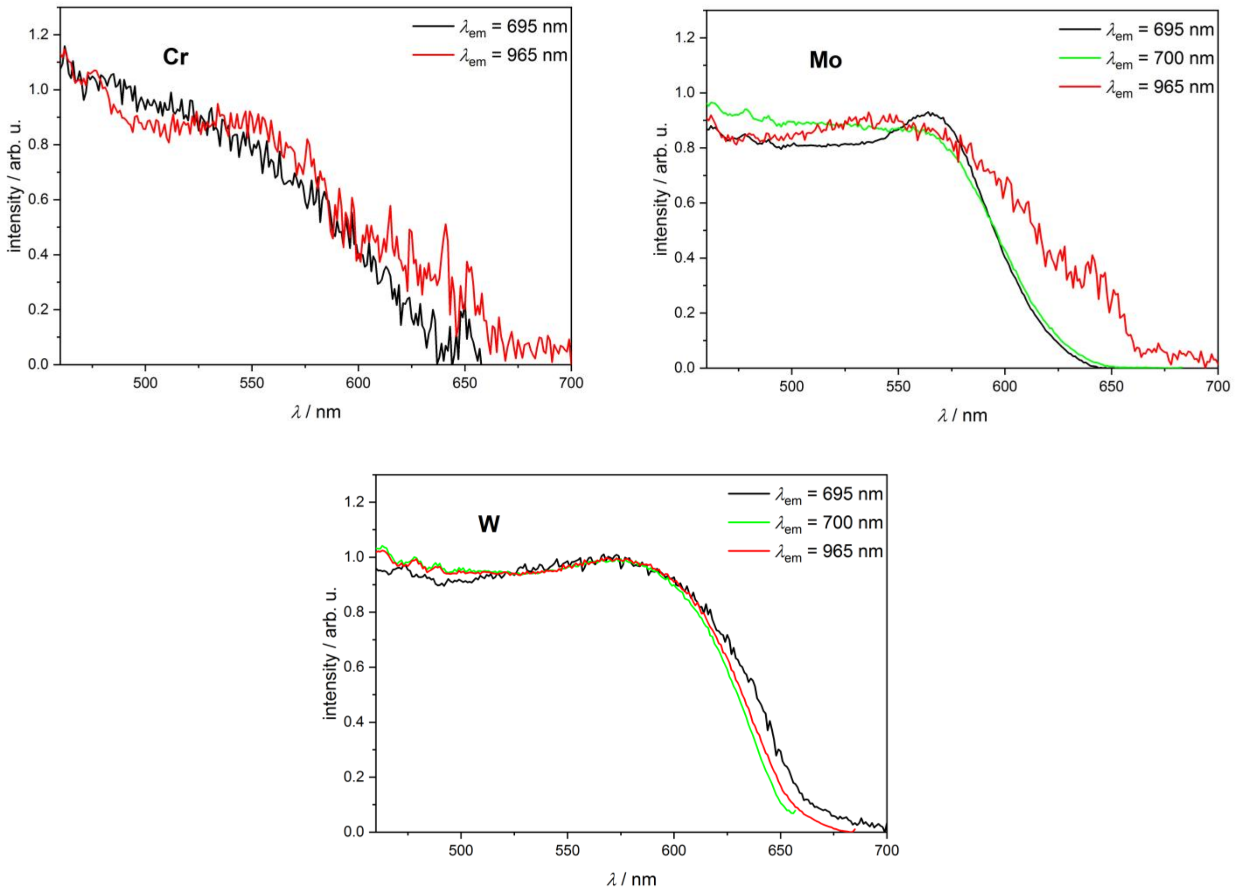

Figure S18. VIS and NIR excitation spectra of **Cr** (top,left), **Mo** (top, right) and **W** (bottom), measured as KBr pellets at room temperature.

6. Static and transient step-scan FTIR spectroscopy

Static FTIR spectra of the solid samples

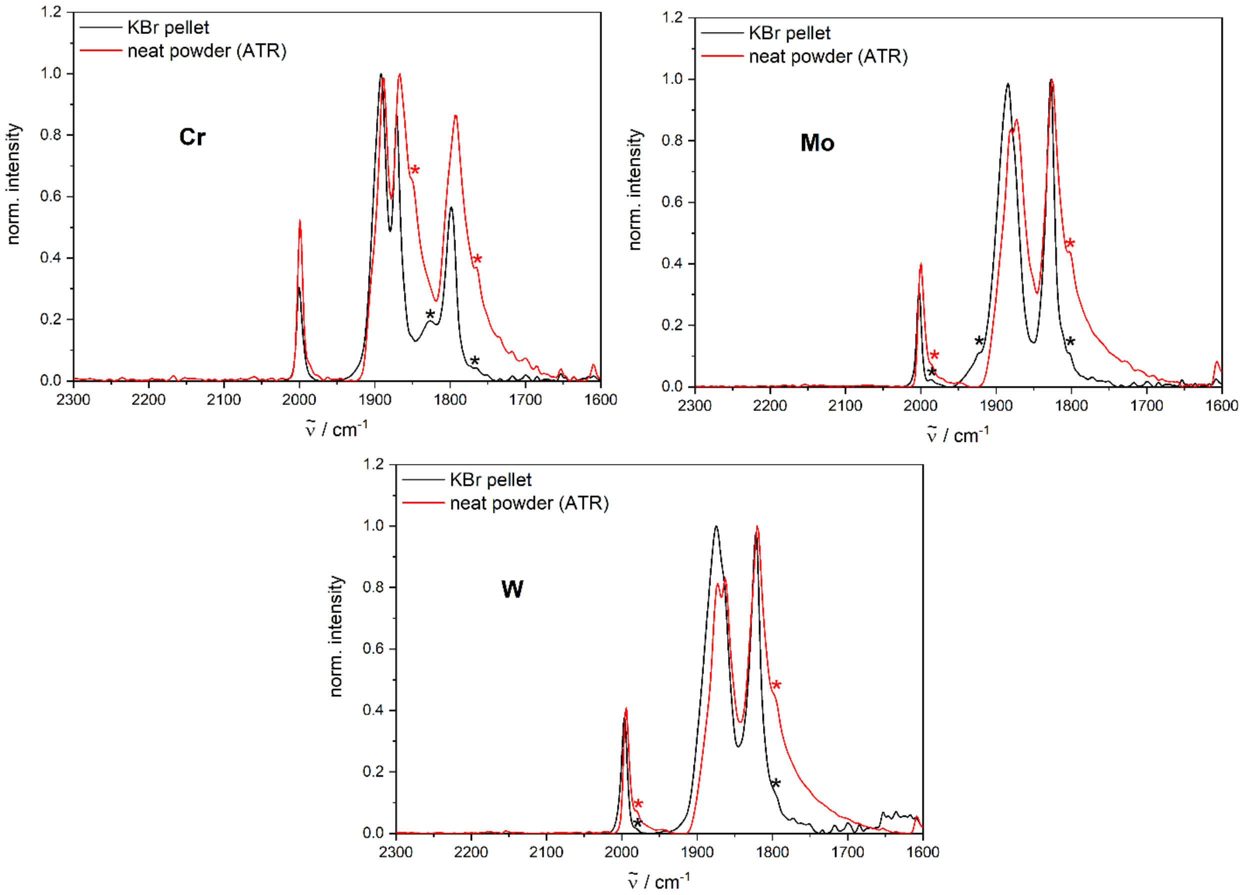

Figure S19. Static solid state IR spectra of **Cr** (top, left), **Mo** (top, right) and **W** (bottom) measured as KBr pellets (black) and neat powders (ATR) (red). The shoulders marked with asterisks are discussed below.

Static IR spectra of solid samples of **Cr**, **Mo** and **W** were recorded as KBr pellets and neat powders (ATR) (Figure S18). The dominating absorption bands in the spectral region of 1700 – 2050 cm^{-1} are assigned to the four CO stretching vibrations. The maxima of these strong peaks are almost identical in the KBr and ATR spectra, so that the influence of the KBr matrix on the CO stretching vibrations is insignificant. The shoulders marked with asterisks in Figure S18 might result from a minor contribution of a second isomer in the solid sample. However, the IR spectrum shows no hint of clearly distinct structures such as dimers or clusters[10], [11], because different structural motifs would probably strongly affect the very sensitive carbonyl stretching vibrations.

General description of step-scan difference spectra

The step-scan difference spectra represent the transient changes of the IR absorption observed after electronic excitation with a laser pulse (λ_{ex} = 532 nm). The negative bands correlate with the vibrational bands in the electronic ground state and result from the depopulation of the electronic ground state. The small deviations between the minima of the negative peaks in the difference spectrum and the absorption maxima in the ground state IR spectrum are explained by the superposition of positive and negative bands in the step-scan difference spectrum. The positive peaks result from the excited state absorption in the populated long-lived electronically excited state(s). The intensity of the step-scan difference spectrum decreases over time due to the repopulation of the electronic ground state. Excited state lifetimes can be obtained from the decay of the transient IR signals (see respective section).

Step-scan difference spectra of **Cr** at 20 K and 290 K

The step-scan difference spectra are averaged over the time range of 0 – 1 µs after laser

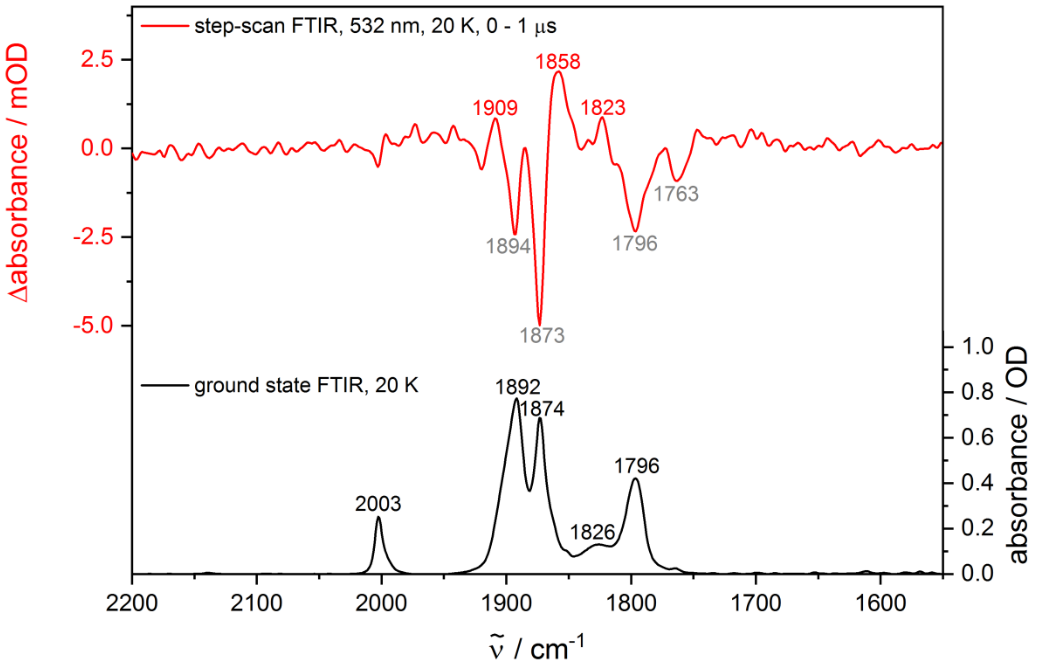

excitation.

Figure S20. Ground state FTIR spectrum and step-scan difference spectrum averaged over 0 – 1 µs after laser excitation (λ_{ex} = 532 nm) of **Cr** (KBr pellet) at 20 K.

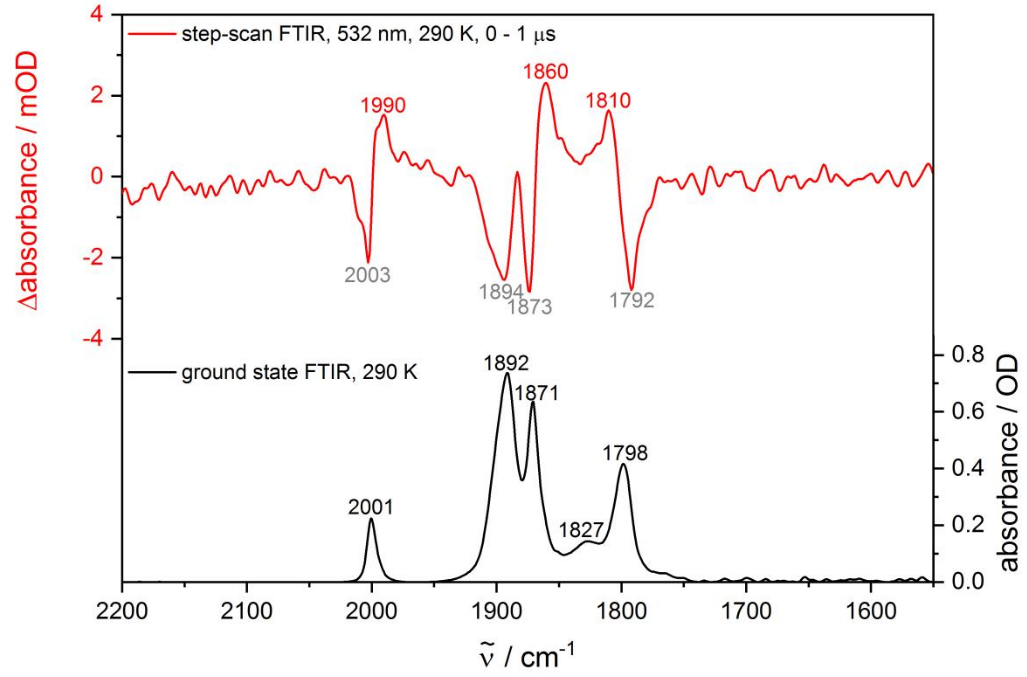

Figure S21. Ground state FTIR spectrum and step-scan difference spectra averaged over 0 – 1 µs after laser excitation (λ_{ex} = 532 nm) of **Cr** (KBr pellet) at 290 K.

Step-scan difference spectra of **Mo** and **W** at 20 K and 290 K

A more remarkable situation is observed when considering the step-scan difference spectra of **Mo** and **W** at 20 K at different times after laser excitation. For these two complexes, the step-scan difference spectra averaged over the first 0.5 µs after laser excitation show strong positive peaks (1977, 1954 and 1934 cm^{-1} for **Mo** and 1943 cm^{-1} for **W**) (Figures S21 and S23), which are not observed at all in the spectra averaged over 5 – 6 µs after excitation. Simultaneously, the other positive and negative features remain clearly recognizable, meaning that two different excited states contribute to the step-scan difference spectrum at short time scales.

At 290 K the positive peaks around 1950 cm^{-1} cited above are hardly visible (**Mo**) or even completely absent (**W**), resulting probably from the shorter excited state lifetimes.

Pure excited state absorption spectra were generated from the presented step-scan difference spectra for a deeper interpretation and comparison with theory (see following section).

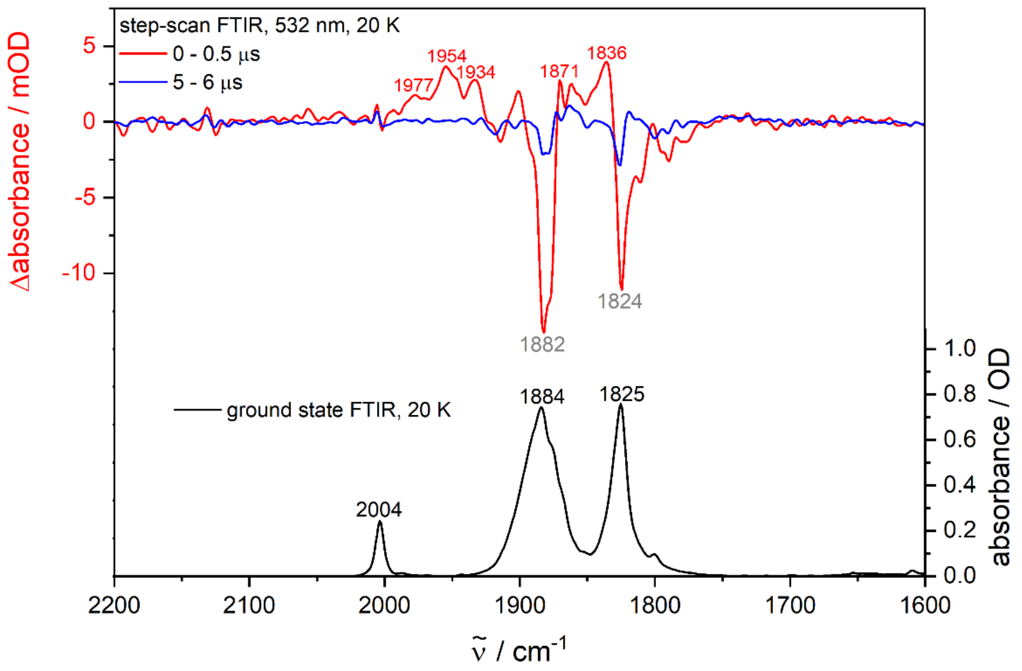

Figure S22. Ground state FTIR spectrum and step-scan difference spectra averaged over 0 – 0.5 µs and 5 – 6 µs after laser excitation (λ_{ex} = 532 nm) of **Mo** (KBr pellet) at 20 K.

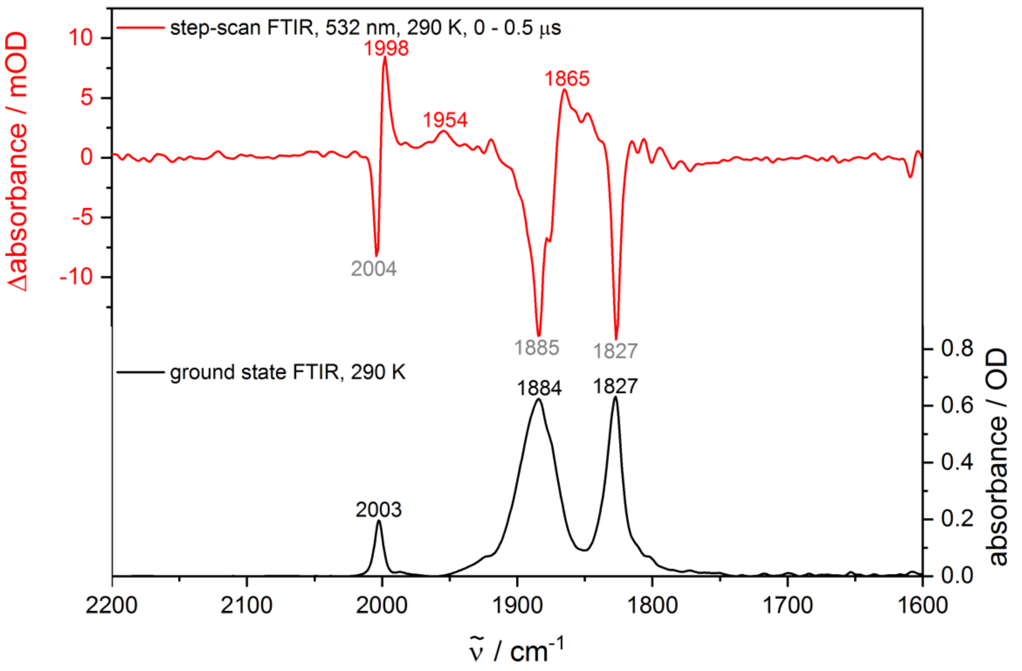

Figure S23. Ground state FTIR spectrum and step-scan difference spectrum averaged over 0 – 0.5 µs after laser excitation (λ_{ex} = 532 nm) of **Mo** (KBr pellet) at 290 K.

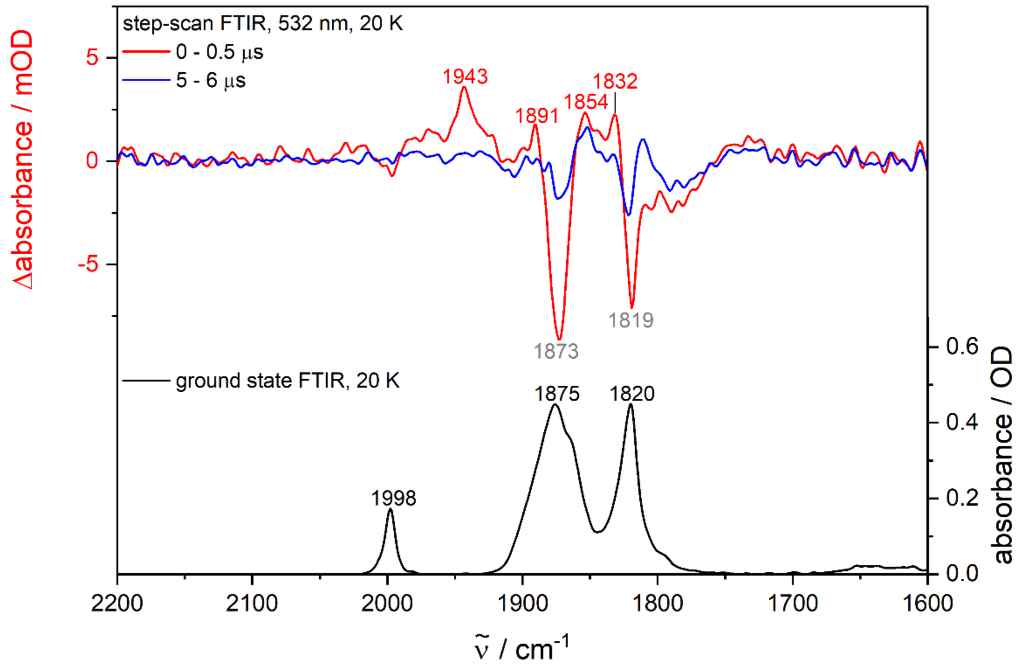

Figure S24. Ground state FTIR spectrum and step-scan difference spectra averaged over 0 – 0.5 µs and 5 — 6 µs after laser excitation (λ_{ex} = 532 nm) of **W** (KBr pellet) at 20 K.

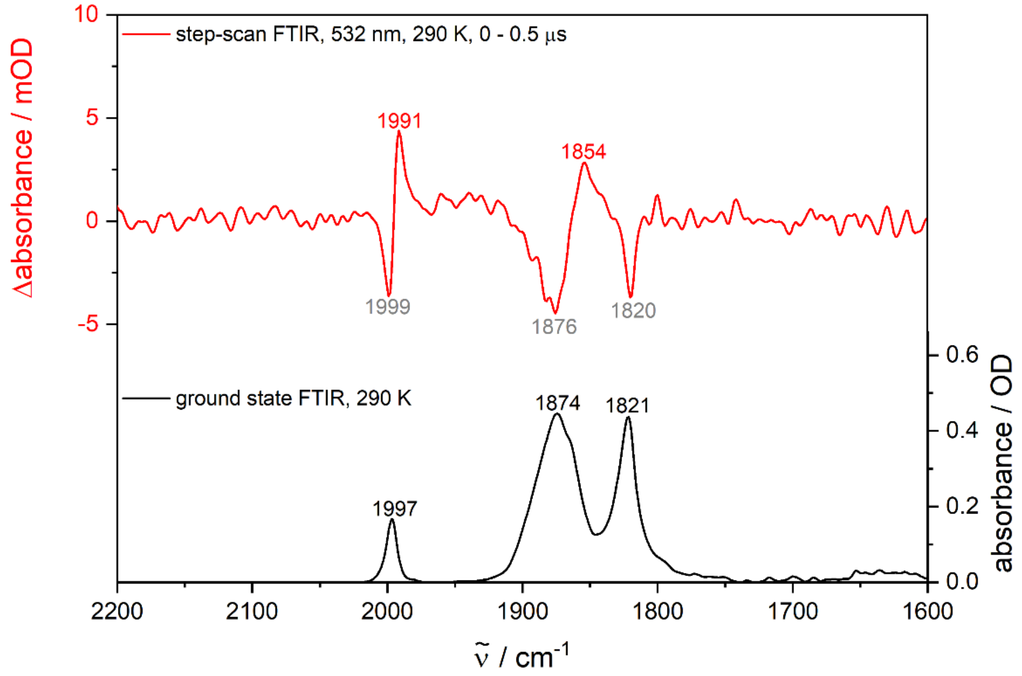

Figure S25. Ground state FTIR spectrum and step-scan difference spectrum averaged over 0 – 0.5 µs after laser excitation (λ_{ex} = 532 nm) of **W** (KBr pellet) at 290 K.

Generation of pure excited state absorption spectra and comparison with theory

The pure IR spectra of the excited states are obtained by addition of a certain contribution of the ground state spectrum to the corresponding step-scan difference spectrum to compensate the negative bands. Contributions of 1.0 – 4.0% of the ground state spectrum were added here, depending on the intensity of the negative bands. The small contribution is explained by the fact that only a small part of the molecules in the pellet is excited by each laser shot.

<u>Excited state absorption spectra of **Cr**</u>

The observation of a weak fifth band beside the four main bands in the excited state absorption spectra of **Cr** at 20 K and 290 K might result from a small contribution of a second isomer to the step-scan difference spectra. Please consider also the discussion on the presence of two isomers in the section on the IR ground state spectra.

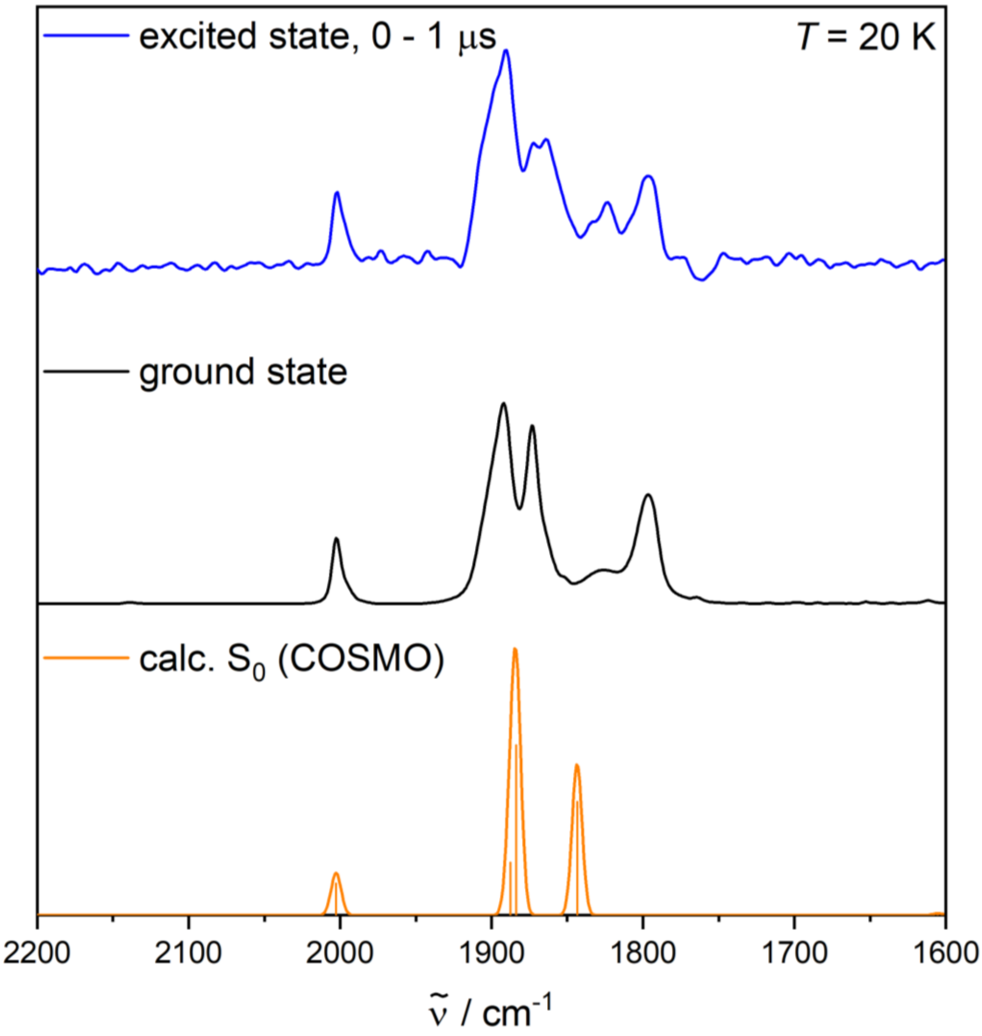

Figure S26. Excited state absorption spectrum obtained from the step-scan spectrum at 0 – 1 µs after laser excitation (top) and ground state FTIR spectrum of **Cr** at 20 K (KBr pellet) (middle) as well as calculated ground state spectrum (S_0) in a KBr matrix (bottom). The sticks represent the calculated IR absorption frequencies. Calculation: DFT/B3LYP-D3(BJ)/def2-TZVP/COSMO, IR absorption frequencies scaled by 0.975, convolution with Gaussian profile, FWHM = 8 cm^{-1}.

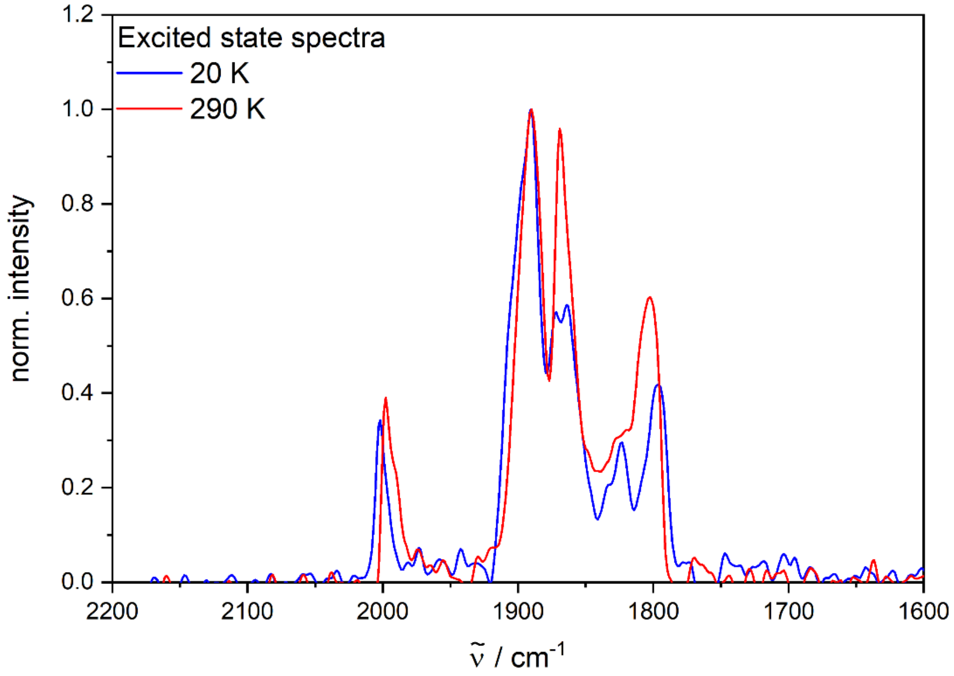

Figure S27. Excited state absorption spectra of **Cr** at 20 K and 290 K (KBr pellet) by considering the step-scan spectra averaged over 0 – 1 µs after laser excitation (λ_{ex} = 532 nm). A contribution of 1.5% of the ground state spectra was added to the step-scan difference spectra.

<u>Excited state absorption spectra of **Mo**</u>

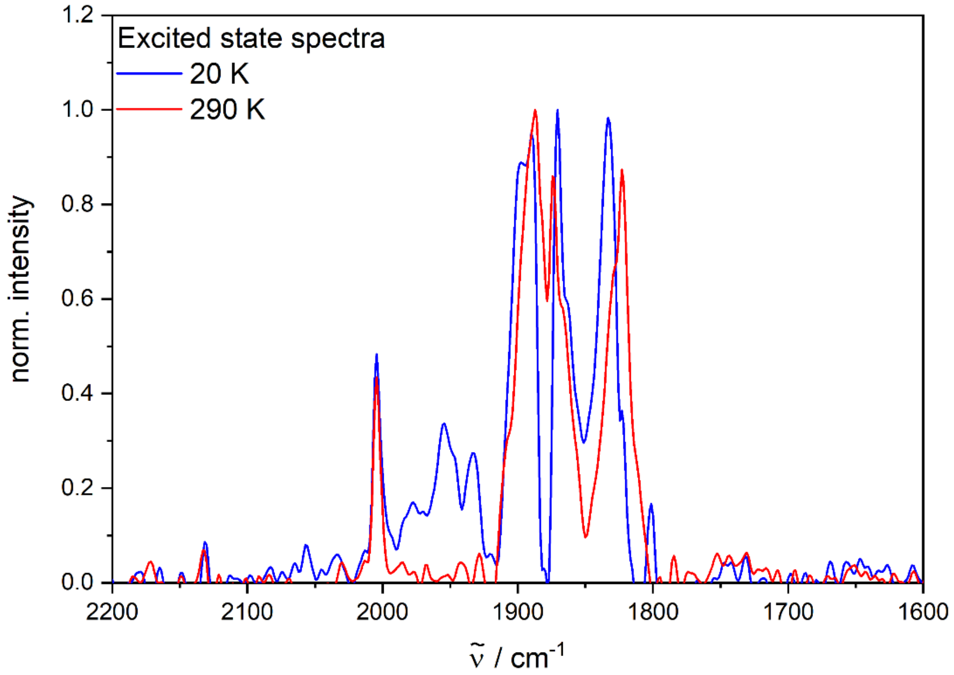

Figure S28. Excited state absorption spectra of **Mo** at 20 K and 290 K (KBr pellet) by considering the step-scan spectra averaged over 0 – 0.5 µs after laser excitation (λ_{ex} = 532 nm). A contribution of 1.0% and 4.0% of the ground state spectrum was added to the step-scan difference spectrum at 20 K and 290 K, respectively.

Excited state absorption spectra of **W**

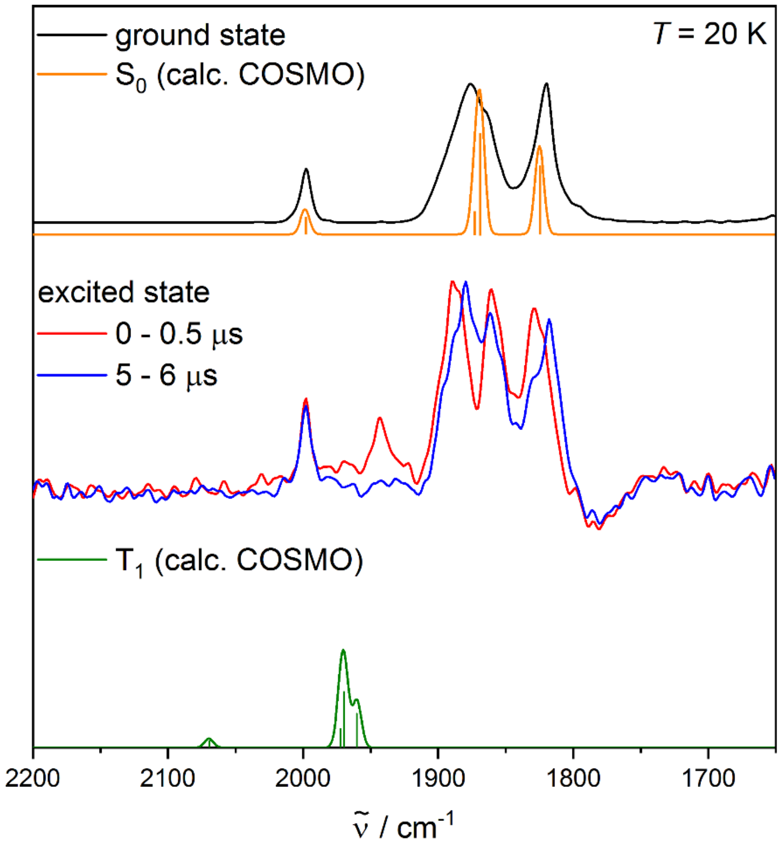

Figure S29. Ground state FTIR spectrum of **W** at 20 K (KBr pellet) and calculated S_0 spectrum in KBr (top), excited state absorption spectra obtained from the step-scan spectra at 0 – 0.5 µs and 5 – 6 µs after laser excitation (middle) as well as calculated triplet (T_n) spectrum in KBr (bottom). The sticks represent the calculated IR absorption frequencies. Calculations: DFT/B3LYP-D3(BJ)/def2-TZVP/COSMO, IR absorption frequencies scaled by 0.975, convolution with Gaussian profile, FWHM = 8 cm^{-1}.

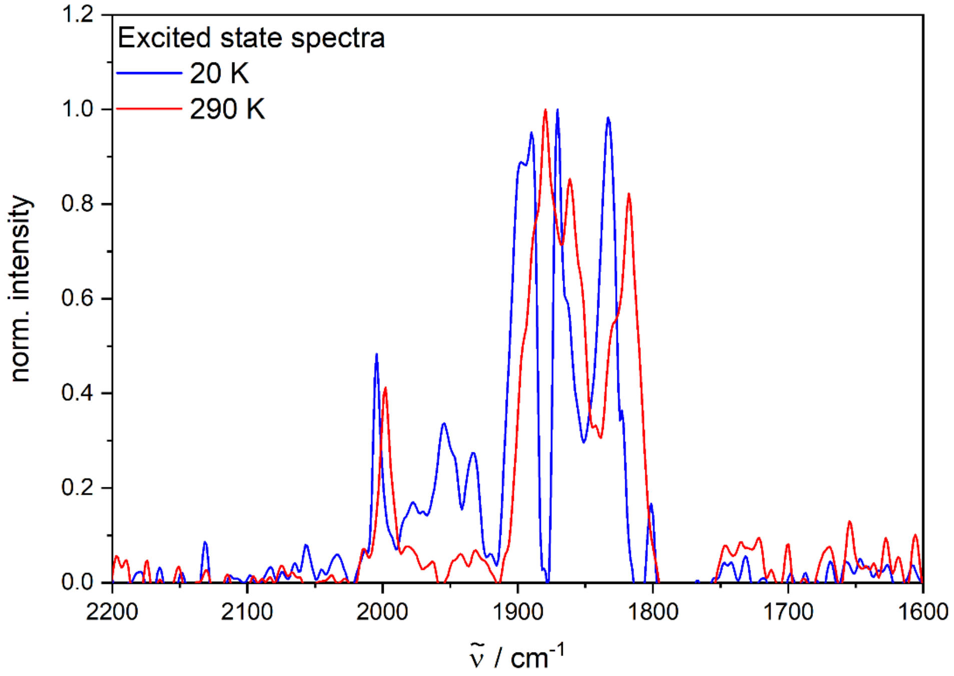

Figure S30. Excited state absorption spectra of **W** at 20 K and 290 K (KBr pellet) by considering the step-scan spectra averaged over 0 – 0.5 µs after laser excitation (λ_{ex} = 532 nm). A contribution of 1.50% and 1.75% of the ground state spectrum was added to the step-scan difference spectrum at 20 K and 290 K, respectively.

Determination of excited state lifetimes by step-scan FTIR spectroscopy

Excited state lifetimes can be obtained from the step-scan data by analysis of the decay of the signals in the step-scan difference spectrum. For this purpose, the most intense bands with the best signal to noise ratio were considered.

<u>Excited state lifetimes of **Cr**</u>

A biexponential decay with two long-lived components on microsecond time scale is observed for **Cr** at 20 K. The shorter time constant with a very small contribution of only 2% might be explained by the presence of traces of a second isomer. Please consider also the discussion on the presence of two isomers in the section on the IR ground state spectra.

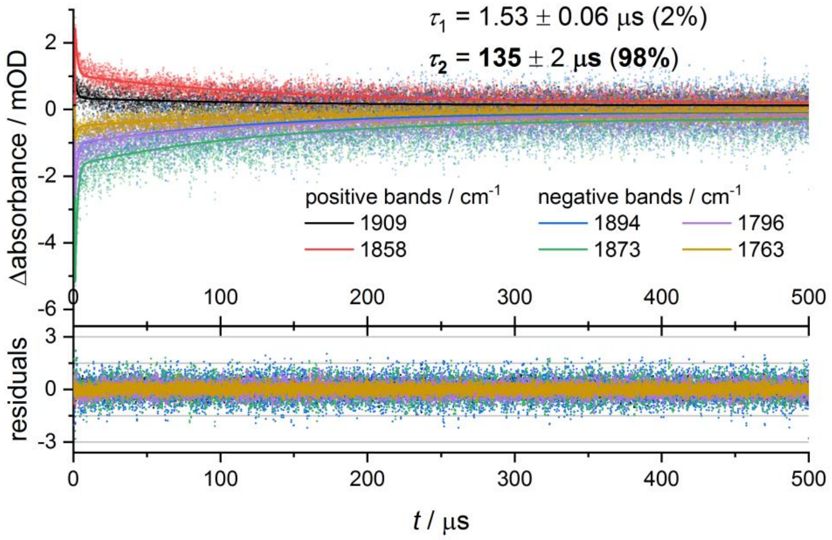

Figure S31. Global biexponential fit performed for the pronounced positive and negative bands in the step-scan spectrum of **Cr** at 20 K.

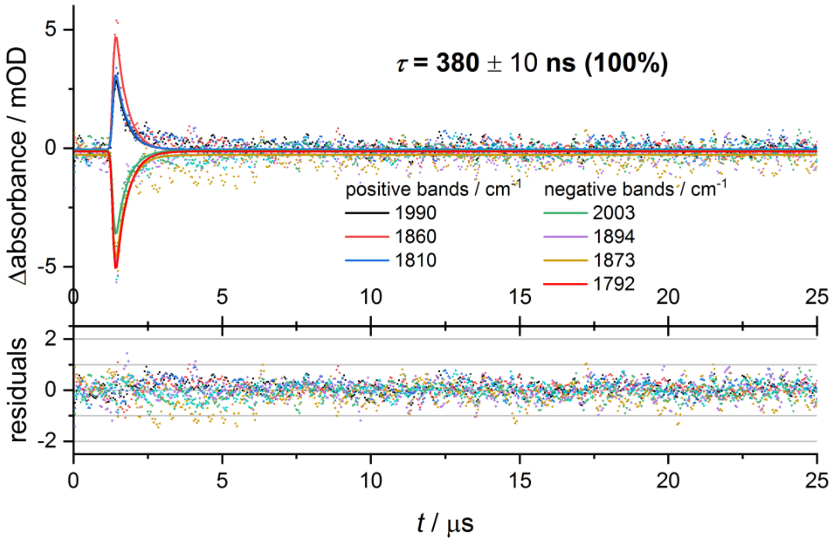

Figure S32. Global monoexponential fit performed for the pronounced positive and negative bands in the step-scan spectrum of **Cr** at 290 K.

Excited state lifetimes of **Mo** and **W**

At 20 K a biexponential fit was performed for the strong negative peaks in the step-scan difference spectrum. This biexponential decay may be explained by the repopulation of the electronic ground state from two distinct excited triplet states (see above and also main text). However, a small contribution of a second isomer cannot be completely excluded, as in the case of **Cr**. Furthermore, a monoexponential fit was performed for the positive peaks between 2000 and 1900 cm^{-1}, assigned to a short-lived excited state. Hence, the lifetime of the short-lived excited state was obtained by averaging the respective time constants from the mentioned mono- and biexponential fits. At the same time, the long-lived component of the biexponential fit is attributed to the excited state with a longer lifetime.

At 290 K a global monoexponential fit was performed by considering all the pronounced positive and negative bands. The obtained time constant is assigned to the NIR emissive triplet state (see main text and above). A reliable determination of the time constants of the short-lived excited state by the step-scan technique was not possible at 290 K due to the weak (**Mo**) or even completely absent features (**W**) of this excited state in the step-scan spectrum at 290 K.

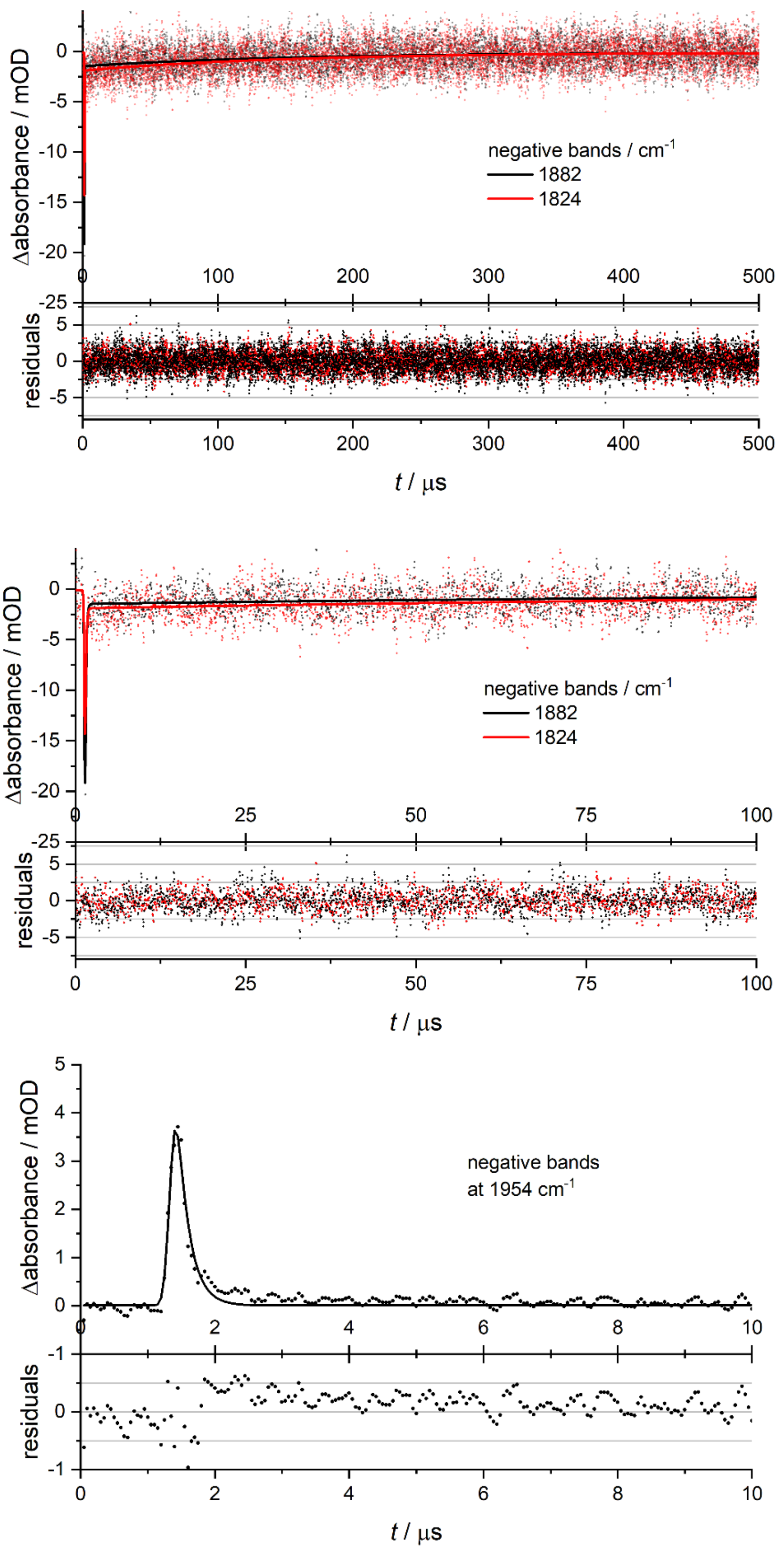

Figure S33. Global biexponential fit performed for the pronounced negative bands in the step-scan spectrum of **Mo** at 20 K (top) and zoom of the time region of 0 — 100 µs (middle). Monoexponential fit for the positive band at 1954 cm^{-1} (bottom).

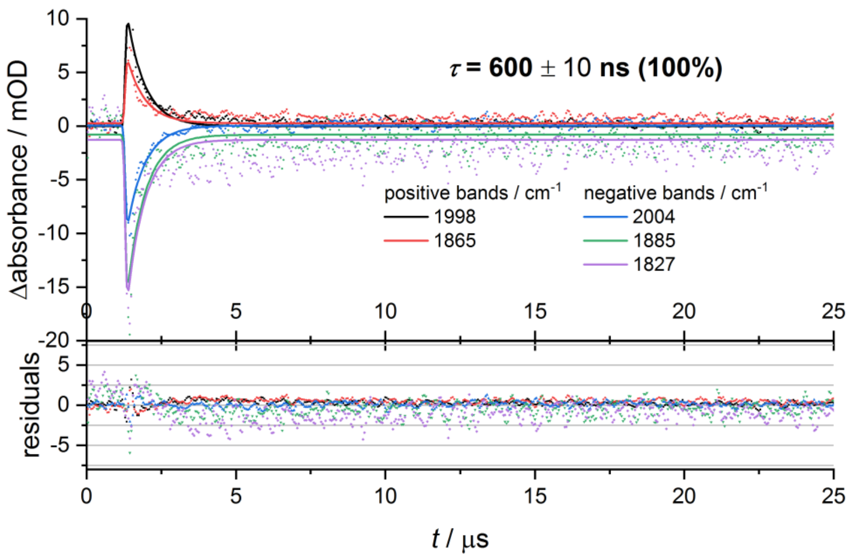

Figure S34. Global monoexponential fit performed for the pronounced positive and negative bands in the step-scan spectrum of **Mo** at 290 K.

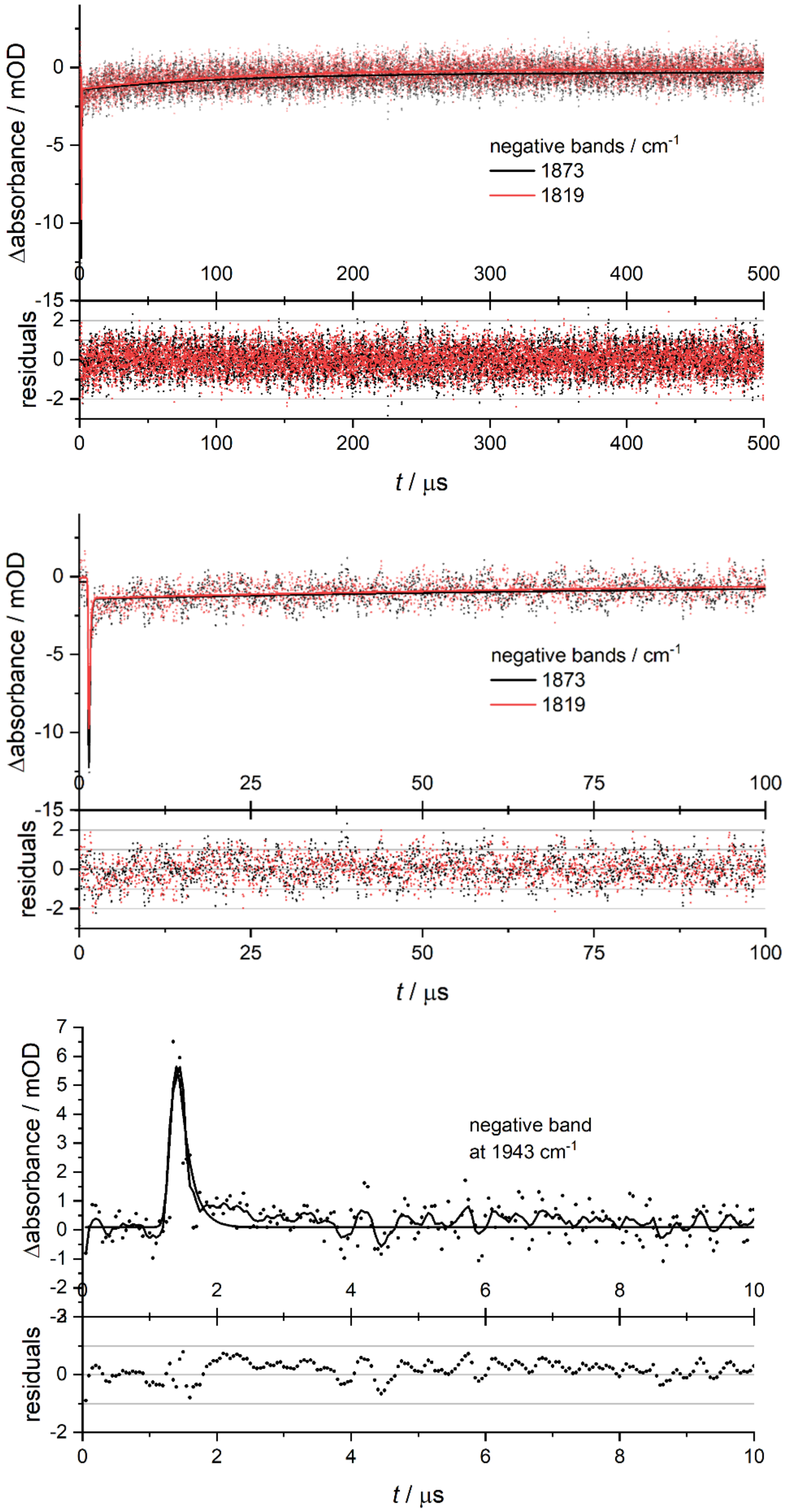

Figure S35. Global biexponential fit performed for the pronounced negative bands in the step-scan spectrum of **W** at 20 K (top) and zoom of the time region of 0 – 100 µs (middle). Monoexponential fit for the positive band at 1943 cm^{-1} (bottom).

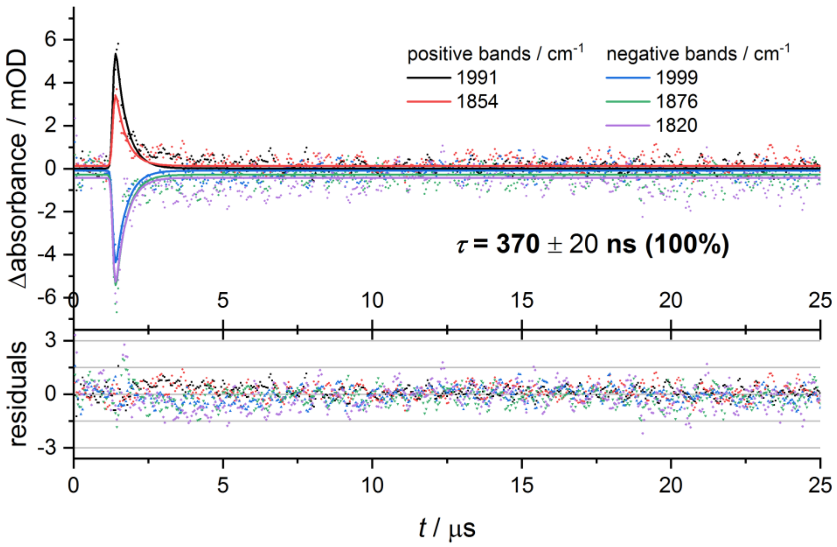

Figure S36. Global monoexponential fit performed for the pronounced positive and negative bands in the step-scan spectrum of **W** at 290 K.

Table S5. Excited state lifetimes of **Cr**, **Mo** and **W** determined by step-scan FTIR spectroscopy.

Complex	290 K	20 K	
	***t* / ns**	**t_1 / µs**	**t_2 / µs**
**Cr**	**380** ± 10 (100%)	**1.53** ± 0.06 (2%)	**135** ± 2 (98%)
**Mo**	**600** ± 10 (100%)	**0.14** ± 0.03 (3%)	**150** ± 10 (97%)
**W**	**370** ± 10 (100%)	**0.17** ± 0.01 (2%)	**116** ± 5 (98%)

7. Additional calculated data

Table S6. Assignment of the calculated electronic excitations in the singlet manifold for Mo in CH_3CN (TDDFT/B3LYP-D3(BJ)/def2-TZVP/COSMO: CH_3CN).

transition	$\tilde{\nu}$ / cm^{-1}	λ / nm	%	donor orbital	acceptor orbital
3	21442		59	HOMO-1	LUMO
		466	40	HOMO-2	LUMO
9	26846	372	91	HOMO-2	LUMO+1
14	33367		41	HOMO-1	LUMO+4
			17	HOMO-4	LUMO
		300	7	HOMO-1	LUMO+3
15	33434	299	78	HOMO-4	LUMO
18	33950		37	HOMO	LUMO+5
			13	HOMO-2	LUMO+7
		295	12	HOMO-2	LUMO+4
30	38308	261	82	HOMO-5	LUMO
33	39769		38	HOMO-1	LUMO+10
			12	HOMO-1	LUMO+7
		251	8	HOMO	LUMO+9
34	40273		21	HOMO-6	LUMO
			15	HOMO-1	LUMO+3
			11	HOMO-2	LUMO+8
		248	11	HOMO-4	LUMO+1
35	40344		39	HOMO-6	LUMO
			19	HOMO-4	LUMO+1
		248	8	HOMO-1	LUMO+3

Table S7. Assignment of the calculated electronic excitations in the singlet manifold for **Mo** in CH_2Cl_2 (TDDFT/B3LYP-D3(BJ)/def2-TZVP/COSMO: CH_2Cl_2).

transition	$\tilde{\nu}$ / cm^{-1}	λ / nm	%	donor orbital	acceptor orbital
3	20562	486	63	HOMO-1	LUMO
			36	HOMO-2	LUMO
8	25885	386	89	HOMO-2	LUMO+1
			5	HOMO-1	LUMO+1
19	33895	295	26	HOMO-2	LUMO+8
			15	HOMO-1	LUMO+3
			14	HOMO	LUMO+6
30	38351	261	80	HOMO-5	LUMO
			6	HOMO-6	LUMO
33	39772	251	27	HOMO-1	LUMO+10
			17	HOMO	LUMO+9
			16	HOMO-1	LUMO+7
34	40068	250	62	HOMO-6	LUMO
			21	HOMO-4	LUMO+1
35	40198	249	21	HOMO-2	LUMO+8
			18	HOMO-1	LUMO+3
			11	HOMO-2	LUMO+2

Table S8. Assignment of the calculated electronic excitations in the singlet manifold for **Mo** in KBr (TDDFT/B3LYP-D3(BJ)/def2-TZVP/COSMO: KBr).

transition	$\tilde{\nu}$ / cm^{-1}	λ / nm	%	donor orbital	acceptor orbital
3	19814	505	65	HOMO-1	LUMO
			33	HOMO-2	LUMO
6	24482	408	80	HOMO	LUMO+2
			17	HOMO-2	LUMO+1
7	25103	398	75	HOMO-2	LUMO+1
			16	HOMO	LUMO+2
20	33930	295	24	HOMO-2	LUMO+8
			21	HOMO	LUMO+7
			15	HOMO-2	LUMO+5
			12	HOMO-1	LUMO+4
31	38401	260	72	HOMO-5	LUMO
			11	HOMO-7	LUMO
33	39637	252	47	HOMO	LUMO+9
			13	HOMO	LUMO+11
			9	HOMO-1	LUMO+7
34	39866	251	65	HOMO-6	LUMO
			17	HOMO-4	LUMO+1
35	40024	250	21	HOMO-2	LUMO+8
			16	HOMO-1	LUMO+4
			13	HOMO-1	LUMO+9
36	40193	249	33	HOMO-1	LUMO+10
			29	HOMO	LUMO+9
			10	HOMO-1	LUMO+7

Calculated molecular orbitals

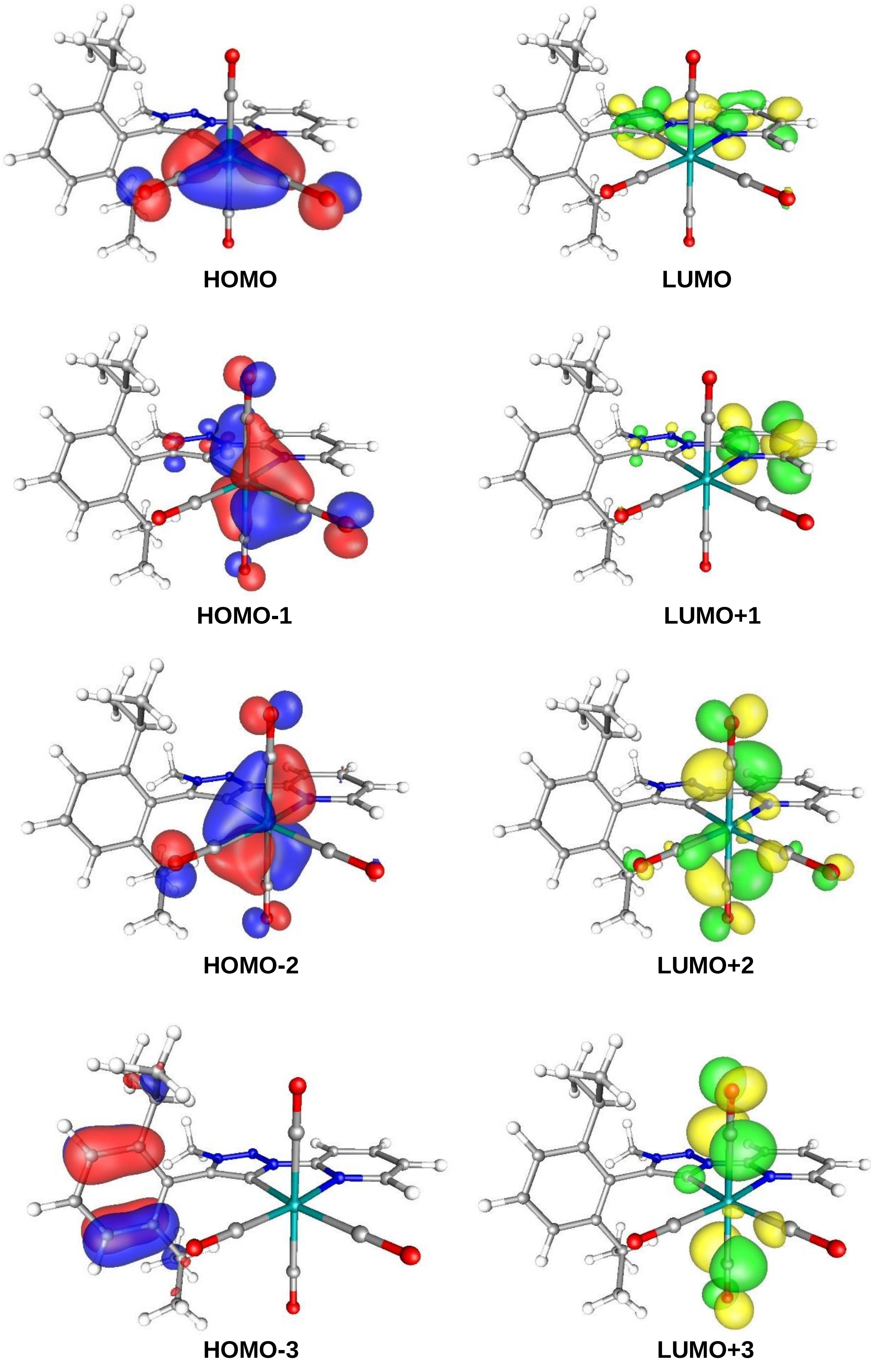

Figure S37. Calculated molecular orbitals of **Mo** in CH_3CN in the electronic ground state S_0 (isovalue: 0.05 a.u.) (DFT/B3LYP-D3(BJ)/def2-TZVP/COSMO: CH_3CN).

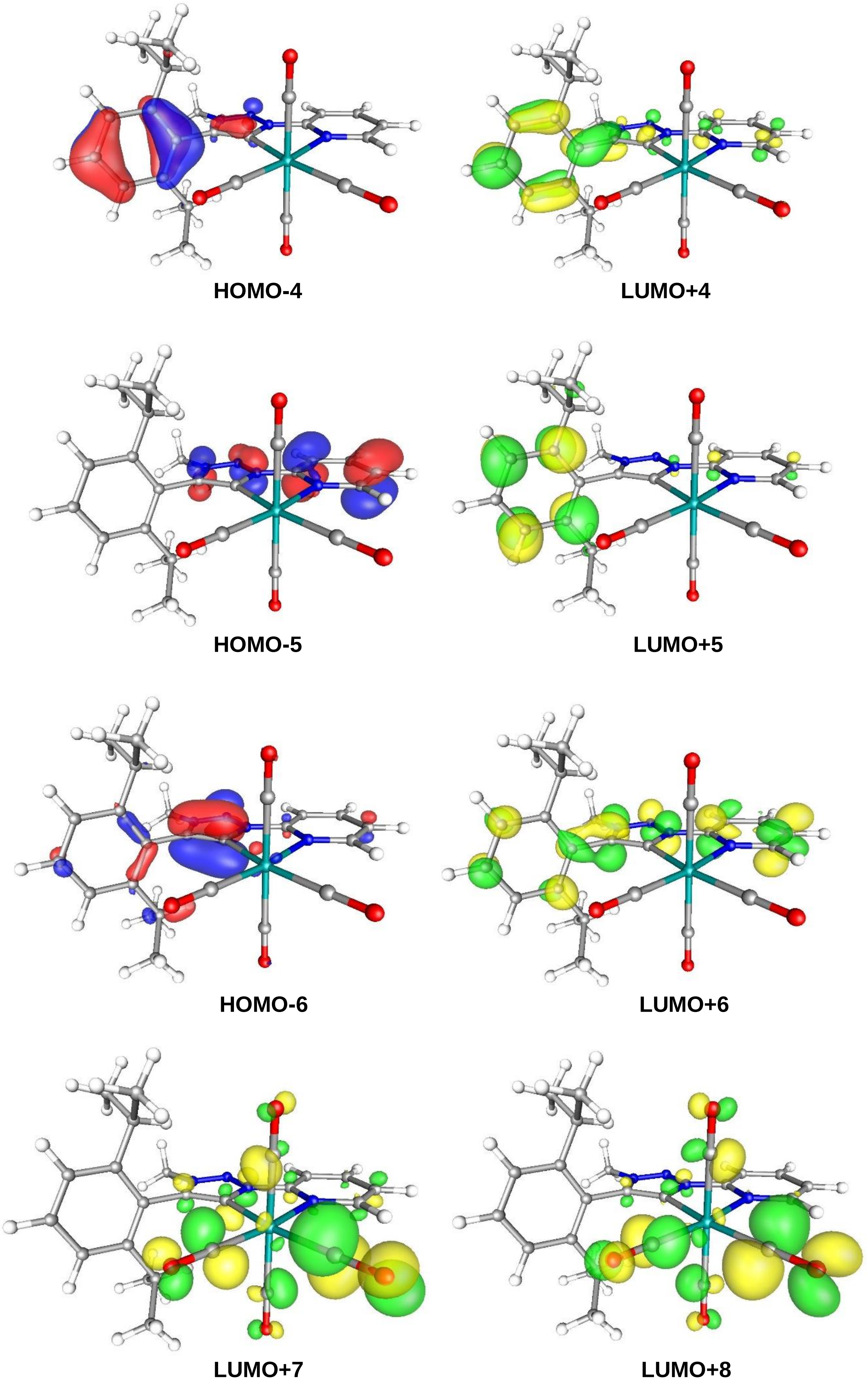

Figure S36. Calculated molecular orbitals of **Mo** in CH_3CN in the electronic ground state S_0 (isovalue: 0.05 a.u.) (DFT/B3LYP-D3(BJ)/def2-TZVP/COSMO: CH_3CN) (continued).

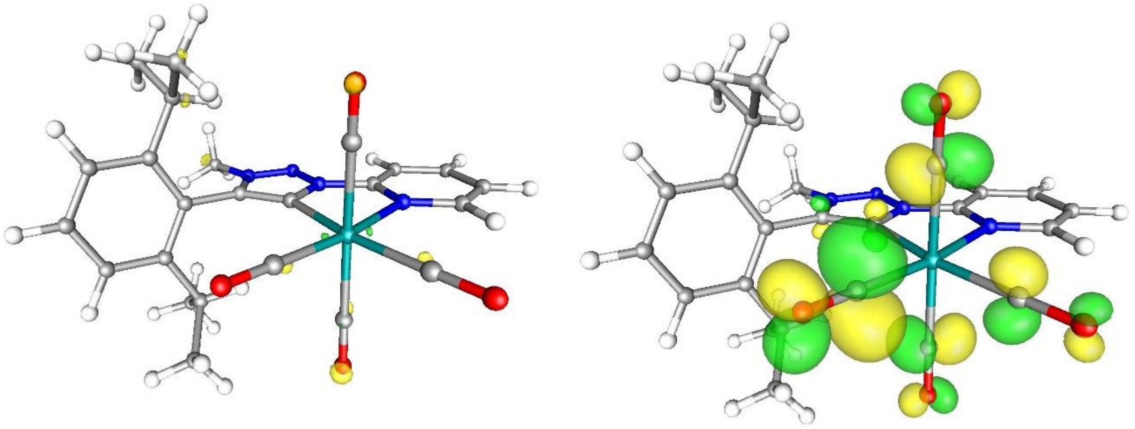

LUMO+9 **LUMO+10**

Figure S36. Calculated molecular orbitals of **Mo** in CH_3CN in the electronic ground state S_0 (isovalue: 0.05 a.u.) (DFT/B3LYP-D3(BJ)/def2-TZVP/COSMO: CH_3CN) (continued).

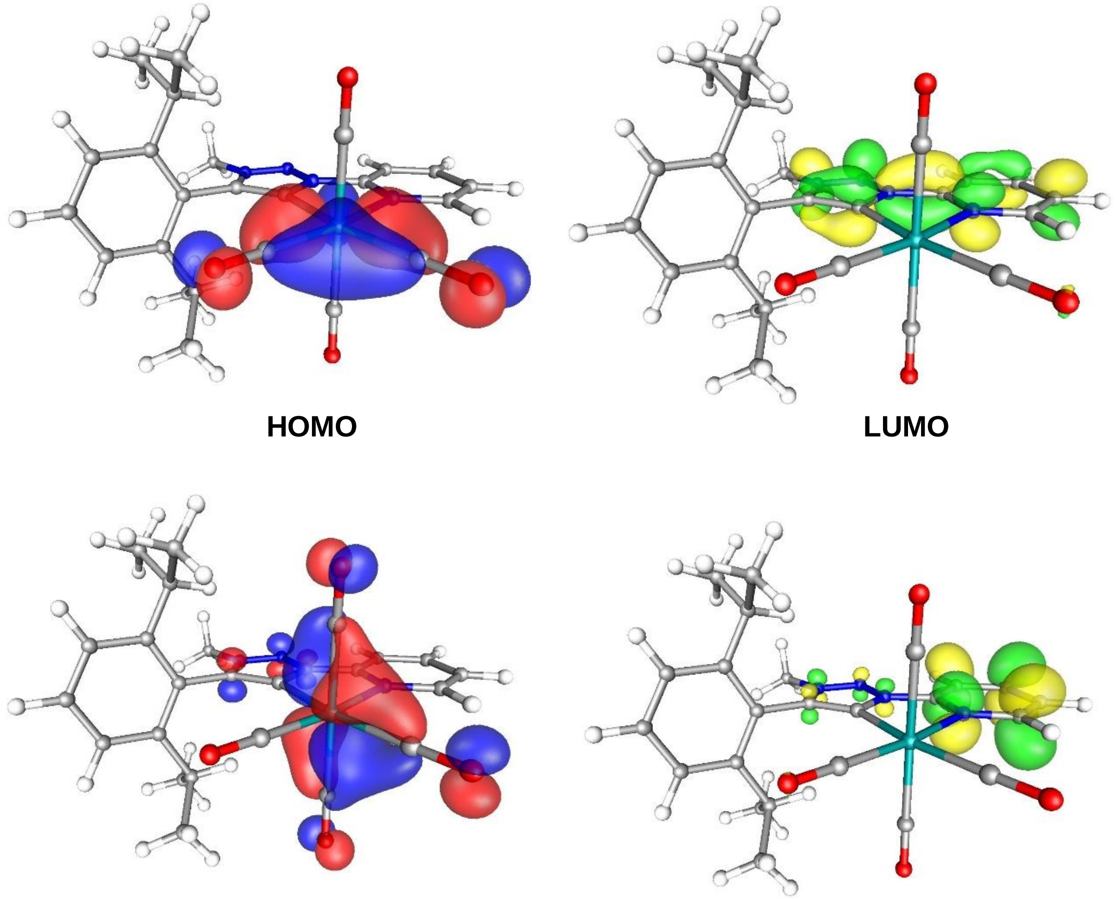

HOMO **LUMO**

HOMO-1 **LUMO+1**

Figure S38. Calculated molecular orbitals of **Mo** in CH_2Cl_2 in the electronic ground state S_0 (isovalue: 0.05 a.u.) (DFT/B3LYP-D3(BJ)/def2-TZVP/COSMO: CH_2Cl_2).

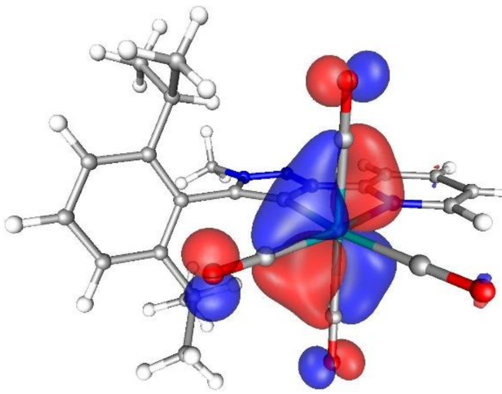

HOMO-2

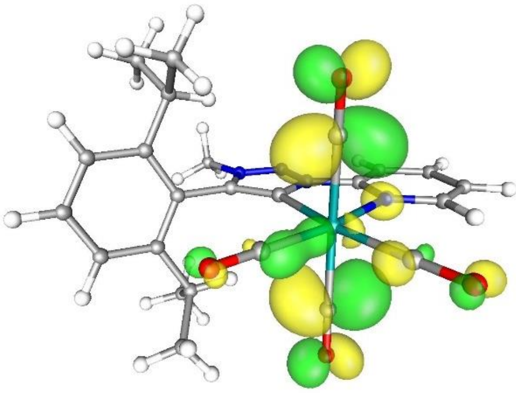

LUMO+2

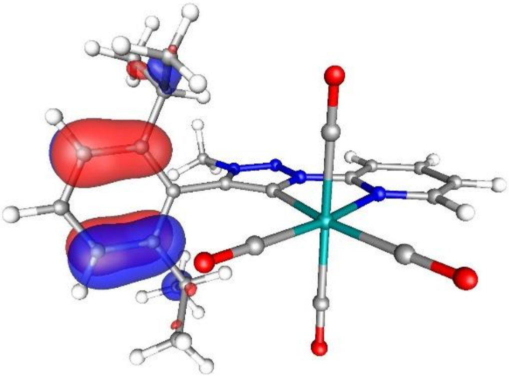

HOMO-3

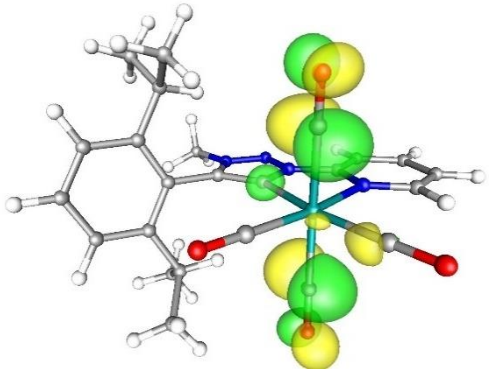

LUMO+3

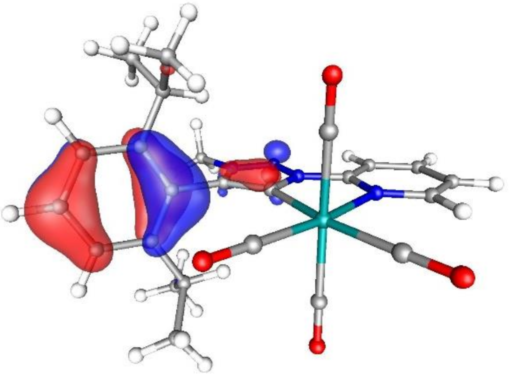

HOMO-4

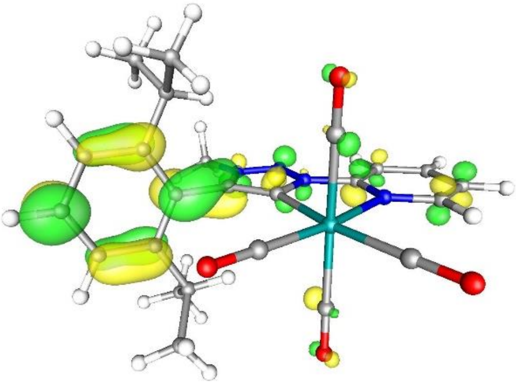

LUMO+4

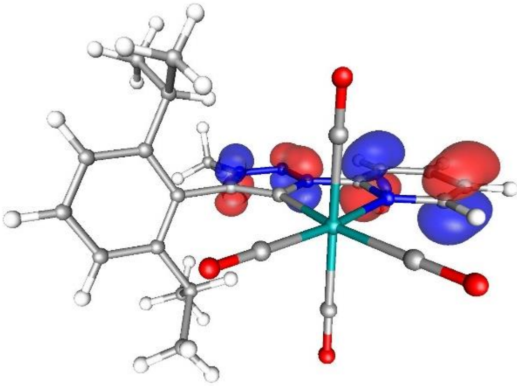

HOMO-5

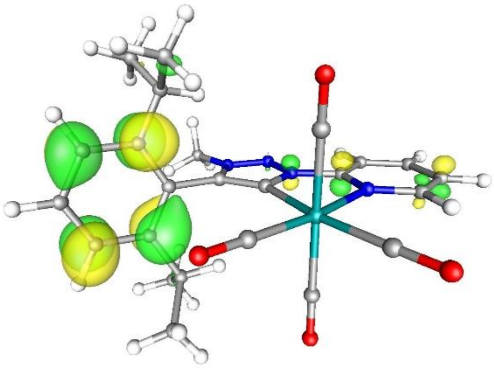

LUMO+5

Figure S37. Calculated molecular orbitals of **Mo** in CH_2Cl_2 in the electronic ground state S_0 (isovalue: 0.05 a.u.) (DFT/B3LYP-D3(BJ)/def2-TZVP/COSMO: CH_2Cl_2) (continued).

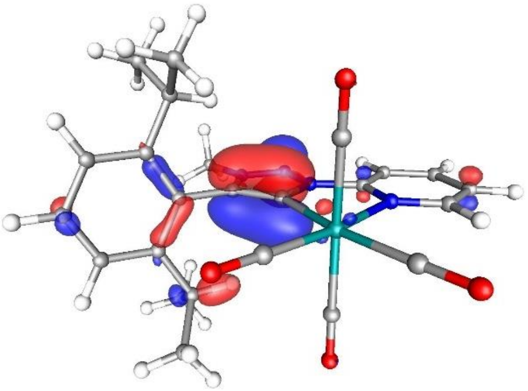

HOMO-6

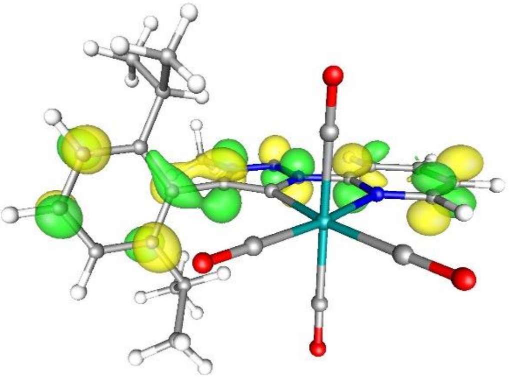

LUMO+6

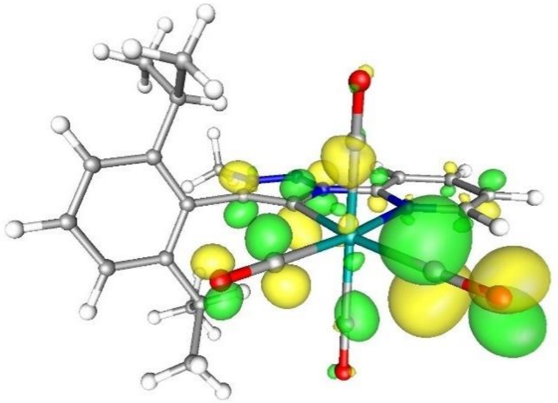

LUMO+7

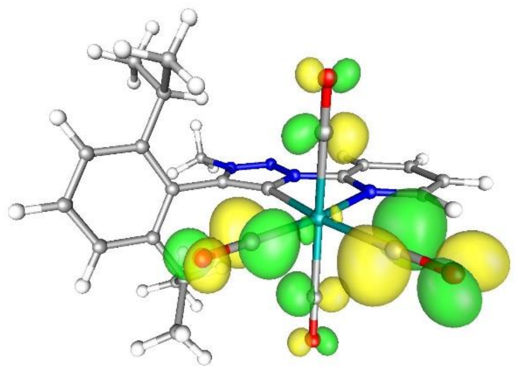

LUMO+8

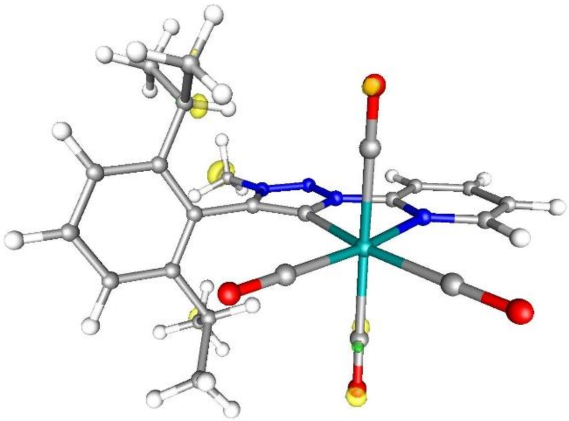

LUMO+9

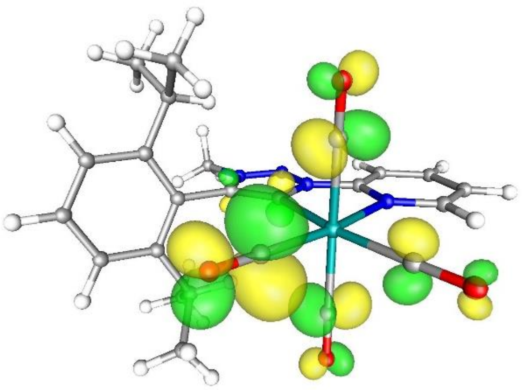

LUMO+10

Figure S37. Calculated molecular orbitals of **Mo** in CH_2Cl_2 in the electronic ground state S_0 (isovalue: 0.05 a.u.) (DFT/B3LYP-D3(BJ)/def2-TZVP/COSMO: CH_2Cl_2) (continued).

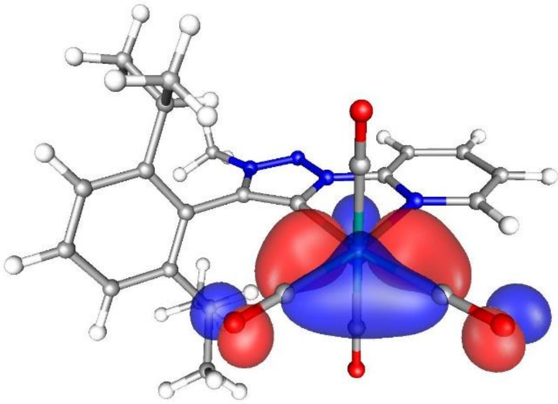

HOMO

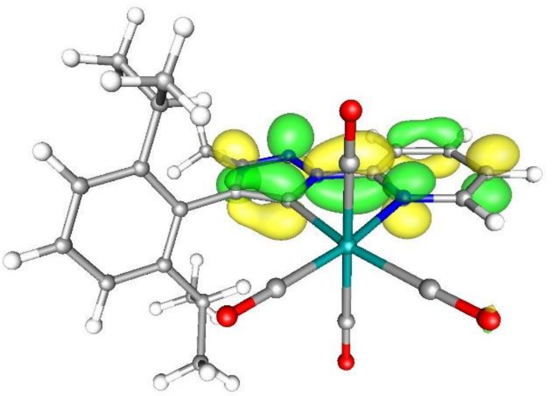

LUMO

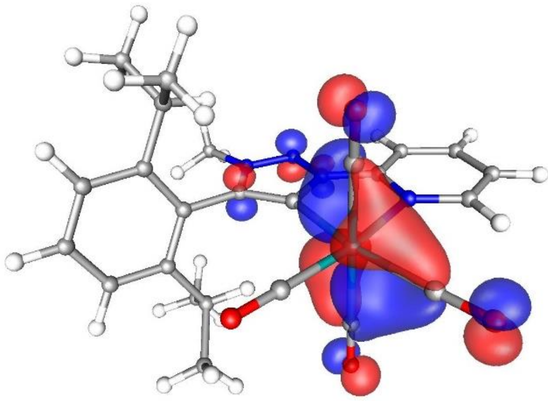

HOMO-1

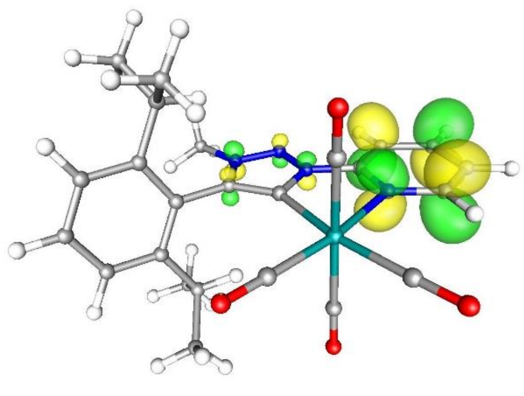

LUMO+1

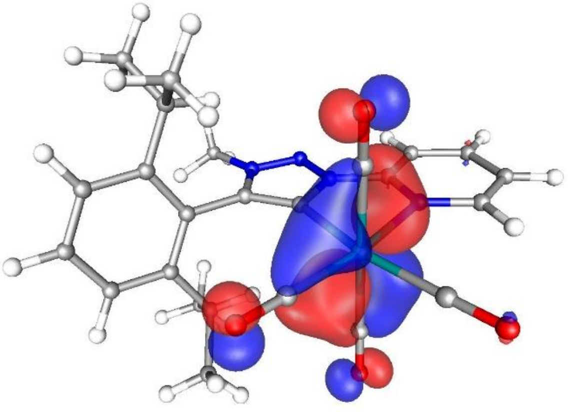

HOMO-2

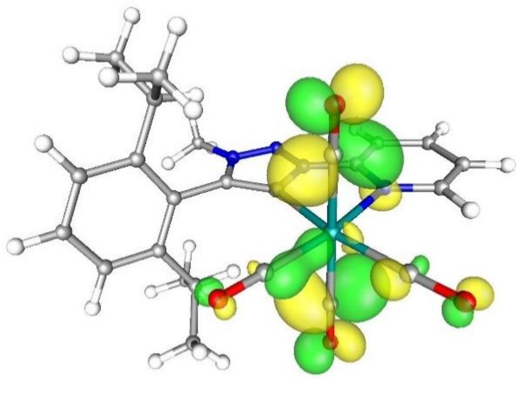

LUMO+2

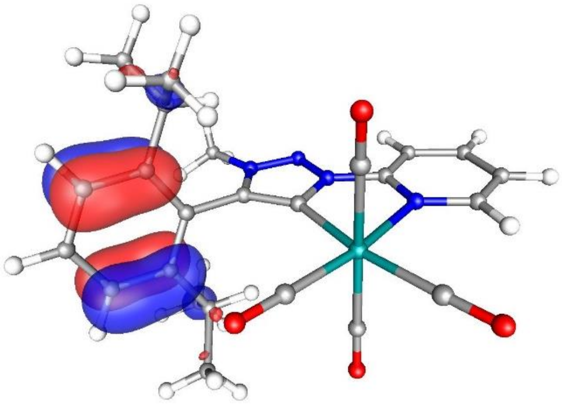

HOMO-3

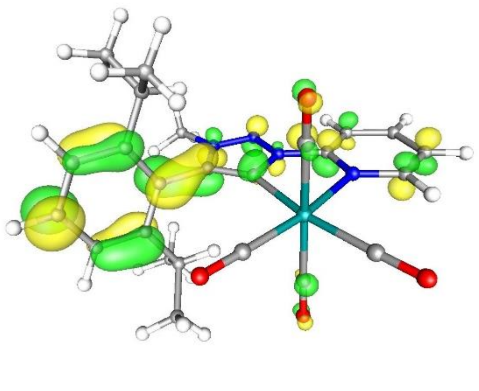

LUMO+3

Figure S39. Calculated molecular orbitals of **Mo** in KBr in the electronic ground state S_0 (isovalue: 0.05 a.u.) (DFT/B3LYP-D3(BJ)/def2-TZVP/COSMO: KBr).

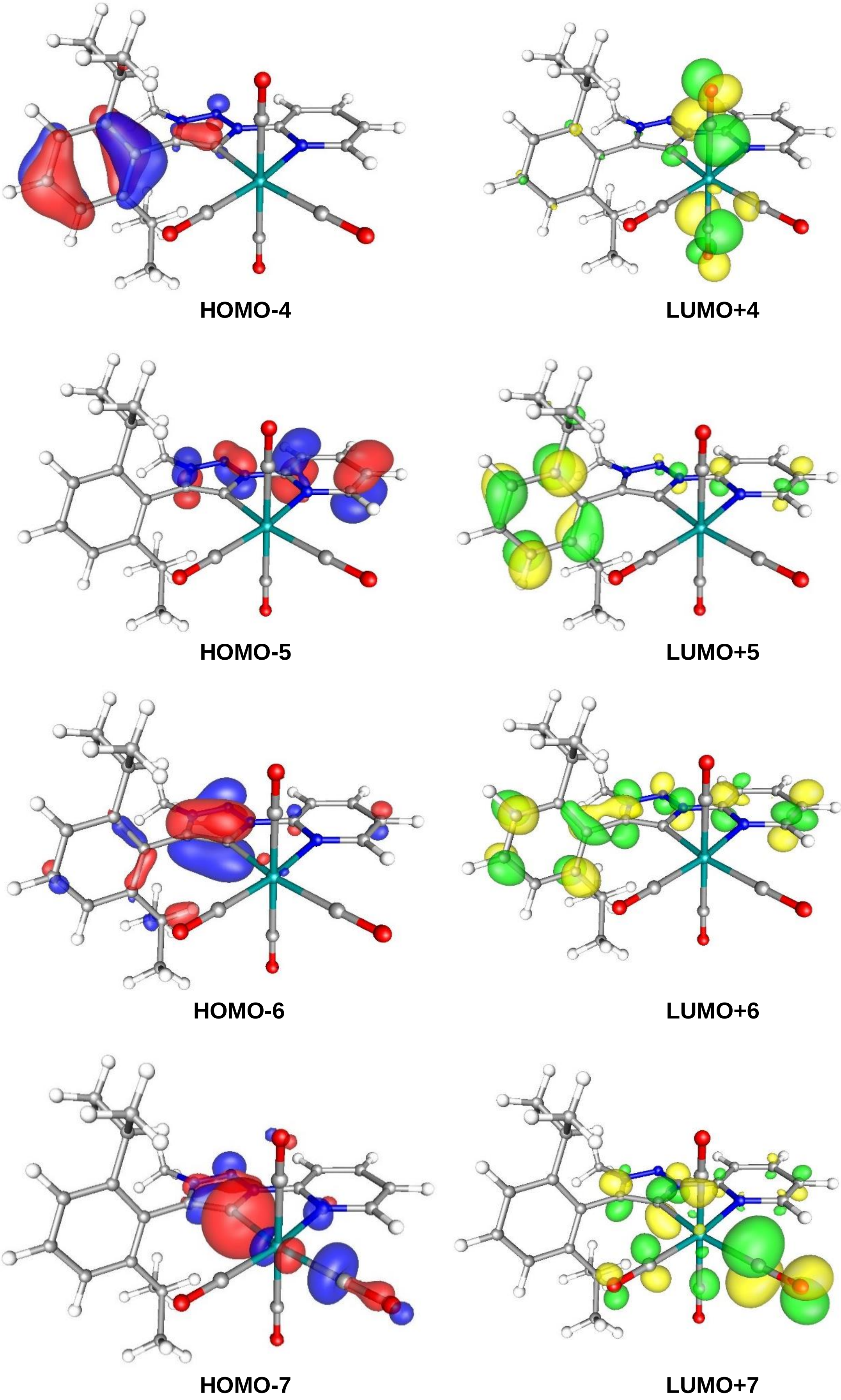

Figure S38. Calculated molecular orbitals of **Mo** in KBr in the electronic ground state S_0 (isovalue: 0.05 a.u.) (DFT/B3LYP-D3(BJ)/def2-TZVP/COSMO: KBr) (continued).

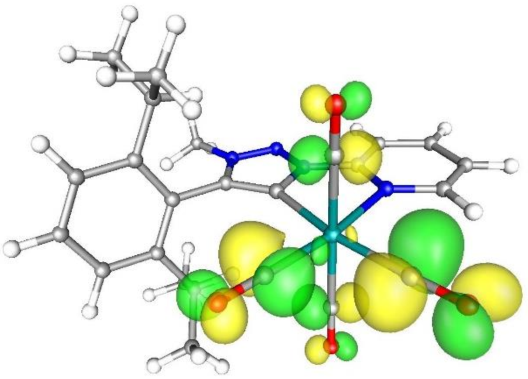

LUMO+8

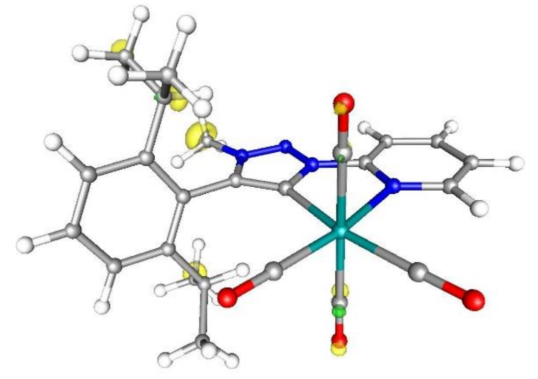

LUMO+9

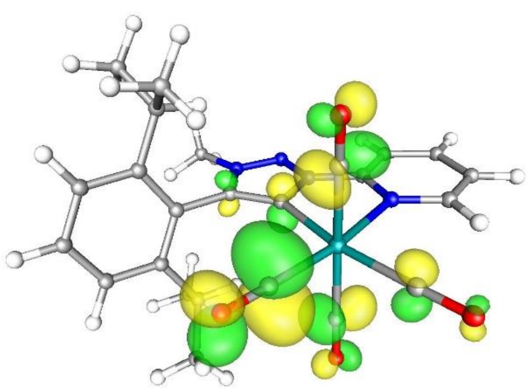

LUMO+10

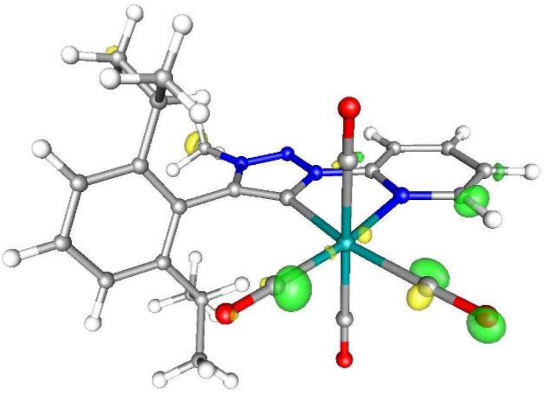

LUMO+11

Figure S38. Calculated molecular orbitals of **Mo** in KBr in the electronic ground state S_0 (isovalue: 0.05 a.u.) (DFT/B3LYP-D3(BJ)/def2-TZVP/COSMO: KBr) (continued).

Spin densities in the triplet state T_n

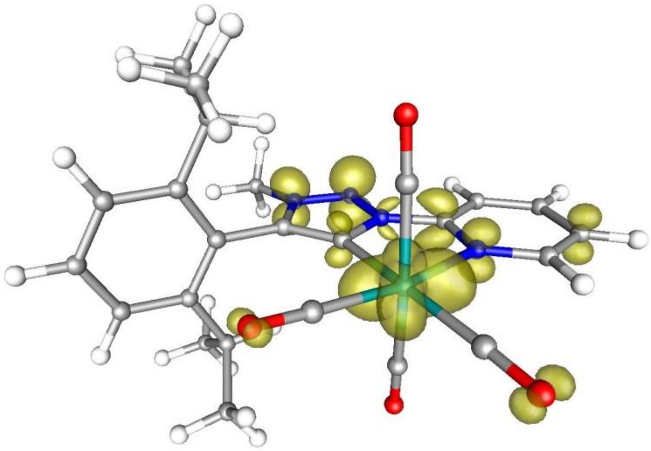

Figure S40. Spin density plot of **Mo** in the triplet state T_n (UDFT/B3LYP-D3(BJ)/def2-TZVP) (isovalue: 0.01 a.u.).

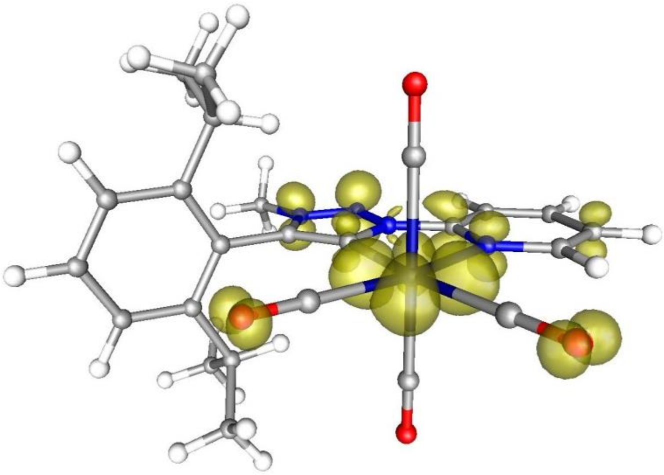

Figure S41. Spin density plot of **W** in the triplet state T_n (UDFT/B3LYP-D3(BJ)/def2-TZVP) (isovalue 0.01 a.u.).

Calculated S_0 and T_n geometries

Table S9. Selected calculated bond lengths [Å] and angles [°] in the ground state (S_0) of **Cr** (DFT/B3LYP-D3(BJ)/def2-TZVP/COSMO: KBr).

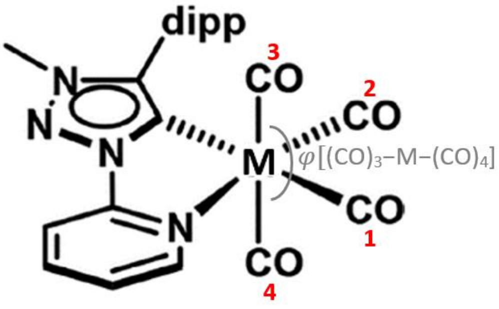

**Cr**	S_0
d(C−O)$_1$	1.160
d(C−O)$_2$	1.159
d(C−O)$_3$	1.151
d(C−O)$_4$	1.151
d(M−CO)$_1$	1.853
d(M−CO)$_2$	1.839
d(M−CO)$_3$	1.894
d(M−CO)$_4$	1.894
d(M−C)	2.264
d(M−N)	2.178
φ[(CO)$_1$−M−(CO)$_2$]	93.4
φ[(CO)$_3$−M−(CO)$_4$]	176.3
φ[C$_{MIC}$−M−N]	76.0

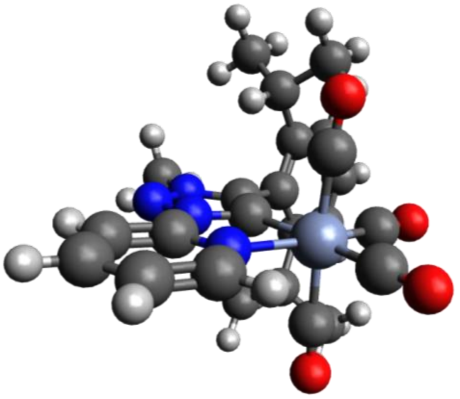

Figure S42. Calculated electronic ground state (S_0) structure of **Cr** in KBr (DFT/B3LYP-D3(BJ)/def2-TZVP/COSMO: KBr).

Table S10. Selected calculated bond lengths [Å] and angles [°] in the electronic ground (S_0) and triplet (T_n) states of **Mo** ((U)DFT/B3LYP-D3(BJ)/def2-TZVP/COSMO: KBr).

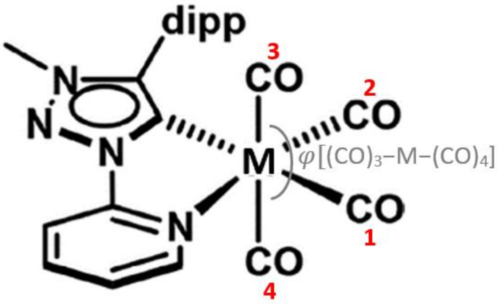

Mo	S_0	T_n	$\Delta(T_1 - S_0)$
$d(C{-}O)_1$	1.160	1.143	-0.017
$d(C{-}O)_2$	1.161	1.143	-0.018
$d(C{-}O)_3$	1.151	1.142	-0.009
$d(C{-}O)_4$	1.151	1.142	-0.009
$d(M{-}CO)_1$	1.997	2.088	+0.091
$d(M{-}CO)_2$	1.966	2.047	+0.081
$d(M{-}CO)_3$	2.044	2.052	+0.008
$d(M{-}CO)_4$	2.043	2.052	+0.009
d(M−C)	2.207	2.191	-0.016
d(M−N)	2.307	2.237	-0.070
$\varphi[(CO)_1{-}M{-}(CO)_2]$	92.9	94.9	+2.0
$\varphi[(CO)_3{-}M{-}(CO)_4]$	172.5	184.8	+12.3
$\varphi[C_{MIC}{-}M{-}N]$	72.3	74.3	+2.0

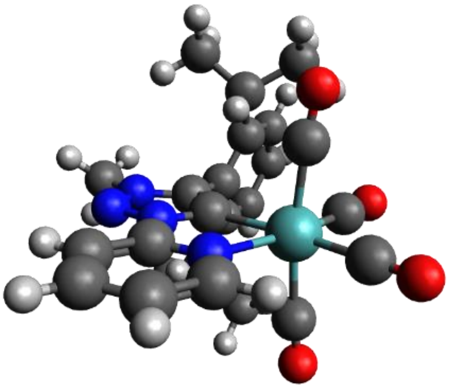

S_0

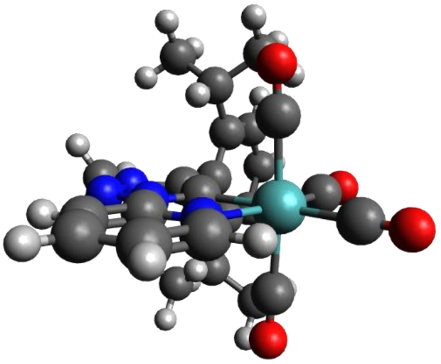

T_n

Figure S43. Calculated electronic ground (S_0) and triplet (T_n) structures of **Mo** in KBr (DFT/B3LYP-D3(BJ)/def2-TZVP/COSMO: KBr).

Table S11. Selected calculated bond lengths [Å] and angles [°] in the electronic ground (S_0) and triplet states (T_n) of **W** ((U)DFT/B3LYP-D3(BJ)/def2-TZVP/COSMO: KBr).

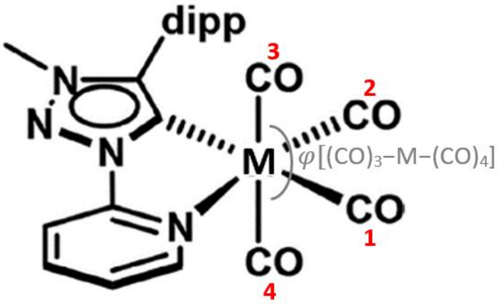

W	S_0	T_1	$\Delta(T_1 - S_0)$
$d(C{-}O)_1$	1.163	1.146	-0.017
$d(C{-}O)_2$	1.163	1.145	-0.018
$d(C{-}O)_3$	1.153	1.144	-0.009
$d(C{-}O)_4$	1.153	1.144	-0.009
$d(M{-}CO)_1$	2.009	2.082	+0.073
$d(M{-}CO)_2$	1.981	2.048	+0.067
$d(M{-}CO)_3$	2.052	2.061	+0.009
$d(M{-}CO)_4$	2.052	2.062	+0.010
$d(M{-}C)$	2.206	2.195	-0.011
$d(M{-}N)$	2.303	2.243	-0.060
$\varphi[(CO)_1{-}M{-}(CO)_2]$	93.5	94.9	+1.4
$\varphi[(CO)_3{-}M{-}(CO)_4]$	171.9	183.3	+11.4
$\varphi[C_{MIC}{-}M{-}N]$	71.9	73.8	+1.9

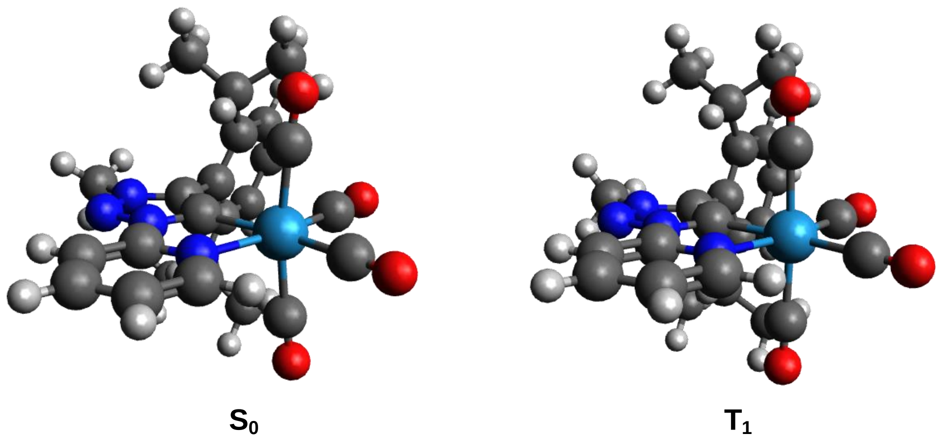

Figure S44. Calculated electronic ground (S_0) and triplet (T_n) structures of **W** in KBr (DFT/B3LYP-D3(BJ)/def2-TZVP/COSMO: KBr).

8. References

[1] M. S. Wrighton, D. S. Ginley, D. L. Morse. *J. Phys. Chem.* **1974**, *78*, 2229.

[2] Y. S. Liu, P. de Mayo, W. R. Ware, *J. Phys. Chem.* **1993**, *97*, 5995.

[3] J. M. Busch, D. M. Zink, P. Di Martino-Fumo, F. R. Rehak, P. Boden, S. Steiger, O. Fuhr, M. Nieger, W. Klopper, M. Gerhards, S. Bräse, *Dalton Trans.* **2019**, *48*, 15687.

[4] S. B. Meshkova, Z. M. Topilova, D. V. Bolshoy, S. V. Beltyukova, *Acta Phys. Pol.* **1999**, *95*, 983.

[5] M. J. Frisch, G. W. Trucks, H. B. Schlegel, G. E. Scuseria, M. A. Robb, J. R. Cheeseman, G. Scalmani, V. Barone, B. Mennucci, G. A. Petersson, H. Nakatsuji, M. Caricato, X. Li, H. P. Hratchian, A. F. Izmaylov, J. Bloino, G. Zheng, J. L. Sonnenberg, M. Hada, M. Ehara, K. Toyota, R. Fukuda, J. Hasegawa, M. Ishida, T. Nakajima, Y. Honda, O. Kitao, H. Nakai, T. Vreven, J. A. Montgomery, Jr., J. E. Peralta, F. Ogliaro, M. Bearpark, J. J. Heyd, E. Brothers, K. N. Kudin, V. N. Staroverov, T. Keith, R. Kobayashi, J. Normand, K. Raghavachari, A. Rendell, J. C. Burant, S. S. Iyengar, J. Tomasi, M. Cossi, N. Rega, J. M. Millam, M. Klene, J. E. Knox, J. B. Cross, V. Bakken, C. Adamo, J. Jaramillo, R. Gomperts, R. E. Stratmann, O. Yazyev, A. J. Austin, R. Cammi, C. Pomelli, J. W. Ochterski, R. L. Martin, K. Morokuma, V. G. Zakrzewski, G. A. Voth, P. Salvador, J. J. Dannenberg, S. Dapprich, A. D. Daniels, O. Farkas, J. B. Foresman, J. V. Ortiz, J. Cioslowski, D. J. Fox, Gaussian 09, Gaussian, Inc., Wallingfort, CT, **2013**.

[6] F. Furche, R. Ahlrichs, C. Hättig, W. Klopper, M. Sierka, F. Weigend, *Wiley Interdiscip. Rev. Comput. Mol. Sci.* **2014**, *4*, 91.

[7] *TURBOMOLE V7.4 2019, a development of University of Karlsruhe and Forschungszentrum Karlsruhe GmbH, 1989-2007, TURBOMOLE GmbH, since 2007; available from http://www.turbomole.com.*

[8] a) S. Grimme, J. Antony, S. Ehrlich, H. Krieg, *J. Chem. Phys.* **2010**, *132*, 154104; b) S. Grimme, S. Ehrlich, L. Goerigk, *J. Comput. Chem.* **2011**, *32*, 1456.

[9] S. Budavari (Ed.) *The Merck-index. An encyclopedia of chemicals, drugs, and biologicals*, Rahway, NJ, **1991**.

[10] O. A. Efremova, M. A. Shestopalov, N. A. Chirtsova, A. I. Smolentsev, Y. V. Mironov, N. Kitamura, K. A. Brylev, A. J. Sutherland, *Dalton Trans.* **2014**, *43*, 6021.

[11] Y. Molard, G. Taupier, S. Paofai, S. Cordier, *Chem. Commun.* **2021**, *57*, 4003.
